# Synthesis of two new lipid mediators from docosahexaenoic acid by combinatorial catalysis involving enzymatic and chemical reaction

**DOI:** 10.1038/s41598-020-76005-5

**Published:** 2020-11-02

**Authors:** Jong-Jae Yi, Sun-Yeon Heo, Jung-Hyun Ju, Baek-Rock Oh, Woo Sung Son, Jeong-Woo Seo

**Affiliations:** 1 Microbial Biotechnology Research Center, Korea Research Institute of Bioscience and Biotechnology (KRIBB), Jeongeup-Si, 56212 Republic of Korea; 2 Department of Pharmacy, College of Pharmacy and Institute of Pharmaceutical Sciences, CHA University, Pocheon-Si, Gyeonggi-do 11160 Republic of Korea

**Keywords:** Biomedical materials, Fatty acids, Small molecules

## Abstract

Omega-3 polyunsaturated fatty acids (PUFAs) have been known to have beneficial effects in the prevention of various diseases. Recently, it was identified that the bioactivities of omega-3 are related to lipid mediators, called pro-resolving lipid mediators (SPMs), converted from PUFAs, so they have attracted much attention as potential pharmaceutical targets. Here, we aimed to build an efficient production system composed of enzymatic and chemical catalysis that converts docosahexaenoic acid (DHA) into lipid mediators. The cyanobacterial lipoxygenase, named Osc-LOX, was identified and characterized, and the binding poses of enzyme and substrates were predicted by ligand docking simulation. DHA was converted into three lipid mediators, a 17*S*-hydroxy-DHA, a 7*S*,17*S*-dihydroxy-DHA (RvD5), and a 7*S*,15*R*-dihydroxy-16*S*,17*S*-epoxy-DPA (new type), by an enzymatic reaction and deoxygenation. Also, two lipid mediators, 7*S*,15*R*,16*S*,17*S*-tetrahydroxy-DPA (new type) and 7*S*,16*R*,17*S*-trihydroxy-DHA (RvD2), were generated from 7*S*,15*R*-dihydroxy-16*S*,17*S*-epoxy-DPA by a chemical reaction. Our study suggests that discovering new enzymes that have not been functionally characterized would be a powerful strategy for producing various lipid mediators. Also, this combination catalysis approach including biological and chemical reactions could be an effective production system for the manufacturing lipid mediators.

## Introduction

Lipid mediators, such as resolvins and protectins, as bioactive signaling molecules are converted from EPA and DHA via lipoxygenase enzymatic reaction at inflamed sites^1^. These lipid mediators actively promote the termination of inflammation with general actions: blocking the conversion of pro-inflammatory mediators such as prostaglandins and leukotriene, releasing other signaling molecules with anti-inflammatory activity, inhibiting the expression of pro-inflammatory cytokines, and promoting macrophage phagocytosis^2,3^. In addition, several in vitro and in vivo studies have demonstrated the pathological effects of SPMs on cancer and vascular diseases^4,5^. Eventually, it was revealed that the health benefits of omega-3 fatty acids in humans are due to the physiological action of their downstream metabolites^6^.

In recent years, lipid mediators behind the beneficial effects of omega-3 fatty acids have attracted much attention as important pharmaceutical targets for the protection and treatment for chronic inflammatory diseases. Consequently, the resolvin E1 analog, RX-10045, is in Phase 2 clinical trials for dry eye and other retinal diseases^7^, and the lipoxin analogs BLXA4 (ZK-142 and ZK-994) are undergoing Phase 1 clinical trials for treatment of inflammatory airway diseases (e.g. asthma)^8^. In contrast to lipid mediators actively promoting the termination of the inflammatory response, traditional steroidal and non-steroidal types of anti-inflammatory drugs have focused on suppressing the initiation of the inflammation. Although conventional medication for inflammation that has been used for a long time can be effective, many of them eventually lead to immunosuppressive which may increase the risk of infection^9^. Hence, resolution therapy based on the SPMs to control various chronic inflammatory diseases may be an innovative therapeutic approach enable to replace current treatments.

Endogenous SPMs potent anti-inflammatory and immune regulatory actions in vivo at very small concentrations in picomolar to nanomolar range^10^. Thus, converting omega-3 fatty acids into high value-added lipid mediators is a very meaningful strategy in the field of lipid industry. To date, most of the commercially available SPMs have been produced by chemical synthesis due to the low activity and stability of the lipoxygenases derived from animals and plants^11–13^. However, chemical synthesis methods also have some demerits, including low yield, toxicity of chemicals, and the time-consuming complicated process. Therefore, it is a very important challenge to develop an eco-friendly and cost-effective in vitro production system using new microbial enzymes that have not been functionally characterized with higher activity capable of manufacturing lipid mediators for commercializing in the cosmetic and pharmaceutical industries.

In the present study, we identified a cyanobacterial lipoxygenase, named Osc-LOX, derived from *Oscillatoria nigro-viridis* PCC 7112, which has high activity and unique activity. Notably, two new types of lipid mediators were generated by combinatorial catalysis consisting of an enzyme-based biological catalyst with a simple chemical catalyst.

## Materials and methods

### Materials

The PUFA substrates LA, AA and EPA were purchased from TCI chemicals (Tokyo, Japan). DHA was obtained from *Thraustochytrid* microalgae (*Schizochytrium* sp. SH103)^14^. The HFA standards 17*S*-HDHA and resolvin D1-5 were obtained from Cayman Chemical (Ann Arbor, MI, USA). The pfu DNA polymerase premix was purchased from Bioneer Inc. (Daejeon, Korea). Restriction enzymes were supplied by New England Biolabs (Beverly, MA, USA). The epoxide hydrolase (EH) was purchased from Sigma-Aldrich (St. Louis, MO, USA). The *E. coli* DH5α strain used for gene cloning was purchased from Real Biotech Corporation (Banqiao, Taiwan). The plasmid pET-28a and *E. coli* BL21 (DE3) were supplied by Novagen (Madison, WI, USA). HiTrap Talon crude (5 mL) and Superdex 200 pg (16/600) were purchased from GE Healthcare (Madison, WI, USA). The SUPELCOSIL LC-DIOL column (25 cm × 3 mm, 5 μm) was obtained from Sigma-Aldrich (St. Louis, MO, USA). The HECTOR-M C18 column (25 cm × 4.6 mm, 5 μm) was purchased from RS Tech (Cheongju, Korea). The CHIRALPAK IB column (25 cm × 4.6 mm, 5 μm) and CHIRALCEL OD-H column (25 cm × 4.6 mm, 5 μm) were obtained from Daicel (Tokyo, Japan). Diaion HP20 resin was obtained from Mitsubishi Chemical (Tokyo, Japan). All solvents for high-performance liquid chromatography (HPLC) analysis were from DaeJung Chemical (Siheung, Korea). All reagents used in this study were extra pure grade.

### Gene cloning

The gene encoding the lipoxygenase, Osc-LOX, from *O. nigro-viridis* PCC 7112 was codon-optimized and synthesized by Bioneer Inc. The synthesized gene was used as a template (Supplementary Fig. S1). The primers used for cloning were designed using SnapGene Viewer software and were based on the sequence of the synthesized gene (Supplementary Table S1). Polymerase chain reaction (PCR) was performed using pfu DNA polymerase premix, and the amplified PCR product was purified using a purification kit. The purified product was inserted between *Nde*I and *Xho*I sites of the pET-28a plasmid, resulting in fusion of a hexa-histidine tag to the N-terminus. The stop codon was located in front of *Xho*I so as to prevent expression of the C-terminal his-tag. *E. coli* strain DH5α was transformed with the constructed plasmids. DNA sequencing, performed by Bioneer Inc., showed that the resulting sequence was identical to the original gene sequence.

### Enzyme over-expression, purification, and measurement of molecular weight

*Escherichia coli* strain BL21 (DE3) was used for target enzyme expression. Host cells harboring pET-28a/*osc-lox* were cultivated at 37 °C in 1-L of Luria–Bertani (LB) medium containing 50 μg mL^−1^ kanamycin using a 5 L flask. After reaching an optical density at 600 nm (OD_600_) of ~ 0.5–0.6, expression of Osc-LOX was induced by adding 0.01 mM isopropyl-β-thiogalactopyranoside (IPTG) and incubating at 20 °C for 24 h on shaking incubator (180 rpm).

Harvested *E. coli* cells containing Osc-LOX were resuspended in buffer A (50 mM Tris–HCl pH 7.5, 500 mM NaCl, 10 mM imidazole, 0.1 mM phenylmethylsulfonyl fluoride, 5% glycerol). The resuspended cells were disrupted by sonication on ice, and then cell debris was removed by centrifugation at 13,000×*g* for 30 min at 4 °C. The supernatant was loaded onto an affinity column equilibrated with buffer A. A cobalt affinity column (HiTrap Talon crude, 5 mL) was utilized in the first step of purification of Osc-LOX. The bound protein was washed with 50 mM imidazole and then eluted with a linear gradient of 10 to 700 mM imidazole at flow rate of 5 mL min^−1^. Subsequently, eluted fractions containing target protein were pooled and concentrated using an Amicon Ultra-15 centrifugal concentrator (10,000 kDa molecular weight cut-off). Concentrated protein was loaded onto a HiLoad 16/600 Superdex 200 pg column (GE Healthcare) that had been previously equilibrated with gel filtration buffer (50 mM Tris–HCl pH 7.5, 150 mM NaCl) and was separated based on its molecular size and shape at a flow rate of 1 mL min^−1^. And, this size-exclusion chromatography (SEC) was used to determine the molecular weight and oligomeric state. The molecular weight of the eluted protein was calculated by comparison of its retention time with that of standard materials. The gel filtration standards, thyroglobulin (670 kDa), γ-globulin (158 kDa), ovalbumin (44 kDa), myoglobulin (17 kDa) and vitamin B12 (1.35 kDa), were used for calibration. Sodium dodecyl sulfate–polyacrylamide gel electrophoresis (SDS-PAGE) on 12% gels was used to confirm purification at each step.

### Enzymatic activity assay and thermal stability

Purified Osc-LOX was used to determine the optimum reaction conditions of pH and temperature toward LA (18:2n-6), as well as the half-life of the enzyme. Unless otherwise noted, all enzymatic reactions for characterization were performed using 3 μg mL^−1^ enzyme and 50 μM substrate for 10 min. The optimum pH for enzyme reactions was determined over a range of pH values from 5.0 to 10.0 using 50 mM 2-(*N*-morpholino) ethanesulfonic acid (MES) buffer (pH 5.0), 50 mM sodium phosphate buffer (pH 6.0–7.0), 50 mM Tris–HCl buffer (pH 8.0) and 50 mM sodium tetraborate buffer (pH 9.0–10.0) at room temperature. The optimum temperature for enzymatic activity and thermal stability was investigated by pre-incubating the reaction mixture without substrate at different temperatures from 20 to 60 °C in 50 mM Tris–HCl buffer (pH 8.0) for 30 min, after which activity was measured following incubation with substrate for 10 min. The half-life of Osc-LOX was measured by incubating the enzyme reaction mixture excluding substrate up to 360 min at a temperature of 30 °C. The enzyme that was continuously incubated at 30 °C was taken every 30 min interval and checked for remaining activity.

### Enzyme kinetics measurements

Arachidonic acid (AA), eicosapentaenoic acid (EPA), and docosahexaenoic acid (DHA) were used to determine the kinetic parameters of purified Osc-LOX. For kinetics analysis, the formation rate of the conjugated diene from the three different substrates was measured by monitoring the increase in absorbance at 234 nm. The enzymatic reaction was performed in a quartz cuvette (10 mm) using 50 mM Tris–HCl buffer (pH 8.0), 3 μg mL^−1^ enzyme, and varying amounts of substrate (25–175 μM) for 5 min at 30 °C. The reaction mixture was quickly mixed, and absorbance was measured every 2 s. Spectra were collected using an Amersham Biosciences Ultrospec 3100 Pro. Data represent the means (± standard deviation, SD) of three separate experiments, each carried out in triplicate. LOX activity was calculated using the extinction coefficient, ε = 23,000 M^−1^ cm^−1^, for the conjugated diene^15^. One unit of LOX activity was defined as the amount of enzyme that catalyzed the formation of 1 μmol of hydroperoxy fatty acid per minute. Specific activity (unit mg^−1^) is defined as the number of units per milligram of protein. Reaction rates were calculated from the initial linear part of the curve. The Michaelis constant (*K*_m_) and turnover number (*k*_cat_) were calculated by nonlinear-regression fits to the Michaelis–Menten equation using the SigmaPlot 10.0 software.

### Enzyme reactions for conversion of DHA into bioactive molecules

Enzymatic reactions were carried out to generate lipid mediators from DHA, by unpurified Osc-LOX (crude enzyme). The DHA as substrate used in the reaction was obtained from *Schizochytrium *sp. SH103^14,16^. The reaction mixture was prepared so as to contain 50 μM substrate in 50 mM Tris–HCl buffer (pH 8.0), and the reaction was initiated by adding Osc-LOX at concentrations of 50–400 units mL^−1^. Reaction mixtures were incubation for 30 min at 30 °C with stirring, after which hydroperoxide products were deoxygenated by adding sodium borohydride to a final concentration of 25 mM. Finally, enzyme reactions were terminated by adding glacial acetic acid (5 μL mL^−1^). The catalyzed products were extracted using an HP20 resin (1/20 of the reaction volume). Briefly, HP20, pre-activated with ethanol, was washed with 5 resin volumes of deionized water. The reaction mixture was loaded onto the cartridge containing HP20 and washed with 10 resin volumes of deionized water and dried under a stream of nitrogen gas. Products bound to HP20 were eluted with 3 resin volumes of ethanol. The extracted eluents were used for HPLC and LC–MS/MS analysis.

### Base catalyzed hydrolysis for epoxide ring-opening

The epoxide ring-opening reaction was carried out using sodium hydroxide (NaOH) as a strong nucleophile. The aqueous solution for the reaction was prepared to contain 0.2 M NaOH in distilled water (DW), to which was added epoxidized material at a final concentration of 100 μg mL^−1^. The reaction mixture was incubated overnight at 30 °C with stirring. As a reference, the hydrolysis reaction for epoxide ring opening was performed using a commercially available EH from *Rhodococcus rhodochrous* (Sigma-Aldrich) in accordance with the manufacturer’s protocol. The hydrolyzed products were extracted using HP20, as described in the enzyme reaction section.

### HPLC analysis for conversion products

HPLC analyses of products converted by Osc-LOX were performed using an Agilent 1200 series equipped with a quaternary pump, solvent degasser, autosampler, thermostatted column compartment, diode array detector, and fraction collector. Normal-phase HPLC (NP-HPLC) was carried out isocratically on a SUPELCOSIL LC-DIOL column (SUPELCO, 25 cm × 3 mm, 5 μm particle size) using heptane/2-propanol/acetic acid (95:5:0.1, by volume) as the solvent. The flow rate was 0.5 mL min^−1^, and the column temperature was maintained at 10 °C. Reversed-phase HPLC (RP-HPLC) was performed on a HECTOR-M C18 column (HECTOR, 25 cm × 4.6 mm, 5 μm particle size) using methanol/water/acetic acid (70:30:0.1 by volume) as a solvent. The reaction products were separated with the mobile phase at a column temperature of 30 °C and a flow rate of 1 mL min^−1^. Enantiomers were separated by normal-phase chiral HPLC (NP-chiral HPLC) using a CHIRALCEL OD-H column (DAICEL, 25 cm × 4.6 mm, 5 μm particle size) and reversed-phase chiral HPLC (RP-chiral HPLC) using a CHIRALPAK IB column (DAICEL, 25 cm × 4.6 mm, 5 μm particle size). For NP-chiral HPLC, a mobile phase consisting of *n*-hexane/2-propanol/acetic acid (95:5:0.1, by volume) was used at a flow rate of 1 mL min^−1^ and a column temperature of 30 °C. For RP-chiral HPLC, a mobile phase consisting of acetonitrile/water/formic acid (50:50:0.1, by volume) was used at a flow rate of 0.8 mL min^−1^ and a column temperature of 25 °C. All samples used for NP-, RP-, NP-chiral and RP-chiral HPLC were dissolved in mobile phase after removing ethanol and were injected in a total volume of 10 μL. Substrate (PUFAs) remaining after the reaction was assessed by monitoring absorbance of samples at 210 nm, and conjugated hydroxy fatty acids were detected by monitoring absorbance at 234 nm, 237 nm, 242 nm, 270 nm, and 302 nm. The standard materials, 17*S*-HDHA, 17*R*-HDHA and resolvin D1-5, were used to identify the reaction products by comparison of retention times. The concentrations of lipid mediators were calculated by correlating peak areas with concentrations of standard materials in linear calibration curves. The calibration curves for standard materials are shown in Supplementary Fig. S2. Crude reaction products were separated by NP-HPLC using a SUPELCOSIL LC-DIOL column, and were collected with a fraction collector. Fractions were evaporated under a stream of nitrogen gas and stored in absolute ethanol at − 80 °C. Purified target compounds were used for NMR analysis.

### LC–MS/MS analysis for identification of conversion products

LC–MS analyses were performed using an Agilent TOF spectrometer (G6550A) with an electrospray ionization (ESI) interface. The converted products produced by enzymatic reactions were separated on a ZORBAX Eclipse Plus C18 Rapid Resolution High Definition column (Agilent, 10 cm × 2.1 mm, 1.8 μm particle size) using an Agilent 6200 series UHPLC system consisting of a binary pump, autosampler, multicolumn thermostat, and diode array detector. The column was eluted isocratically with a solvent of water/acetonitrile/formic acid (90:10:0.1, by volume) at a column temperature of 40 °C and a flow rate 0.3 mL min^−1^. All samples used in analyses were dissolved in absolute ethanol after removing methanol and were injected in a total volume of 2 μL. LC flow was directly injected into a mass spectrometer equipped with a Dual AJS ESI source. Sample ionization was performed in negative mode using the following conditions: drying gas, nitrogen (14 L min^−1^, 200 °C); nebulizer gas, nitrogen (35 psi); capillary voltage, 3.5 kV; capillary temperature, 350 °C. Spectra were recorded over an *m/z* range of 50–3000 with an accumulation rate of 2 spectra/s. Data were processed using Mass Hunter Workstation Acquisition software. The chemical structure was drawn and the molecular masses of its fragments were calculated using ChemDraw Professional v 15.1.

### Nuclear magnetic resonance (NMR) spectroscopy

NMR experiments were conducted using a Bruker Avance III HD spectrometer (800 MHz) with a 5 mm triple resonance inverse (TCI) Cryo Probe at the Research Institute of Pharmaceutical Sciences (Seoul National University, Seoul, Republic of Korea). Purified lipid mediators, prepared at a concentration of 5 to 7 mg mL^−1^ were dissolved in methanol-d4 (CD_3_OD) and measured at 298 K. In addition to 1D NMR (^1^H, ^13^C), the following 2D NMR experiments were performed: correlation spectroscopy (COSY), total correlation spectroscopy (TOCSY), nuclear Overhauser effect spectroscopy (NOESY), hetero-nuclear single-quantum correlation spectroscopy (Edited-HSQC), and hetero-nuclear multiple-bond correlation spectroscopy (HMBC)^17^. NMRPipe^18^ and NMRView software^19^ were employed for NMR data processing and peak assignment. All samples were confirmed by several characteristic proton and carbon resonances. Protons were assigned using integrated ^1^H-COSY (1D ^1^H and 2D ^1^H) spectra, which measure the correlation between adjacent protons. Then, protons were assigned to carbons using 2D-Edited-HSQC (^1^H and ^13^C) spectra, which record single-through-bond relationships. TOCSY and HMBC spectra were used to identify relationships through either two or three bonds, and to distinguish overlapping peaks. Stereochemistry were confirmed by NOESY spectra based on the identified stereo information of the precursor.

### Polarimetric analysis of conversion products

Specific rotations were measured using a 10 mL cell with 100 mm path length on a JASCO P-2000 Digital Polarimeter. All conversion products were prepared in concentrations ranging from 0.05 to 0.1 mg mL^−1^ in ethyl alcohol, and their specific rotations were detected in absorbance at 589 nm at room temperature.

### Molecular modeling and substrate docking simulation

The 3D structure of Osc-LOX was acquired using known crystal structures, soybean LOX-3 (PDB ID: 1NO3 and 1HU9), with high sequence homology (21% identity) as templates^20,21^. Initial three-dimensional (3D) structures were generated using the I-TASSER method^22,23^. The generated structure of Osc-LOX was then refined by performing loop modeling using a modeler loop-building algorithm implemented in UCSF Chimera 1.11.2 (https://www.cgl.ucsf.edu/chimera/)^24,25^. Finally, energy minimization of modeled structure was performed after clean up and addition of hydrogen using YASARA 19.5.5 (https://www.yasara.org/)^26–28^. Molecular surface analyses of protein structures and calculation of electrostatic surface potentials (ESP) were investigated using UCSF Chimera and YASARA. The structures of lipid mediators were drawn using MarvinSketch 17.28 (2018, ChemAxon, https://www.chemaxon.com), and 3D structures of lipid mediators were generated and minimized using UFF/GAFF force field in Avogadro^29^ and Spartan 18^30^. Molecular docking of Osc-LOX with lipid mediators was performed using AutoDock Vina^31,32^, and analyses of ligand–protein interactions were performed using PLIP (Protein–Ligand Interaction Profiler)^33^. All figures of structures were generated with UCSF Chimera and YASARA.

### Database search

The protein database UniProt was used to search the sequence of lipoxygenases. The database tools LIPID MAPS Lipidomics Gateway, METLIN, ChemSpider, and PubChem were used to confirm the identity of lipid mediators based on the results of LC–MS/MS and NMR analyses.

## Results and discussion

### Conserved sequence analysis

The sequence of *osc-lox* (accession number K9VMV7, 1713 bp) was found in the UniProt database for gene synthesis; however, its enzymatic activity studies had not been reported. The *osc-lox* gene encodes a protein consisting of 571 amino acids (calculated molecular mass, 64.9 kDa). A sequence-alignment analysis was performed with conserved sequences of previously reported LOX enzymes from animals, plants, fungi, proteobacteria, and cyanobacteria (Supplementary Fig. S3). The metal-binding amino acids in all LOXs were perfectly conserved as the motif, HHHN(H/S)I; however, Osc-LOX incorporates a methionine in the last position instead of isoleucine. Alanine and glycine in the catalytic site of LOX are preserved and known as stereo-controlled determinants called the Coffa site^34^. In the Coffa site hypothesis, alanine is conserved in S-type LOX and glycine is conserved in R-type LOX; Osc-LOX contains alanine 296, and thus is an S-type LOX.

### Preparation of Osc-LOX for enzyme assay and reaction

Osc-LOX containing a His^6^-tag had a molecular weight (theoretically calculated) of approximately 67.2 kDa (Supplementary Fig. S4a, left). Cobalt affinity chromatography was utilized as a first step of purification. Osc-LOX was eluted over an imidazole concentration range of 170 to 190 mM (Supplementary Fig. S4a, middle). To obtain highly purified enzyme, we performed size-exclusion chromatography (SEC). Osc-LOX was retained on the SEC column for 82 min, and the relative molecular weight was confirmed to be ~ 67.5 kDa (Supplementary Fig. S4b). Approximately 15 mg of Osc-LOX purified with high purity of 96% was obtained from a 1-L culture (Supplementary Fig. S4a, right). Since the enzyme was concentrated, the specific activity (unit mg^−1^) of the enzyme increased at each purification stage, but the total activity decreased owing to loss of protein during the purification process. Total activity (unit, U) of unpurified Osc-LOX (crude enzyme) was calculated to be 146 kilounits. After the first purification step, 71.23% (104 kilounits) of the activity remained, and after the second purification step, 27.4% (40 kilounits) of the activity remained (Supplementary Table S2). Total activity refers to the amount of enzyme that can convert monohydroperoxy fatty acids from DHA. The highly purified Osc-LOX was used for characterization and kinetics assays. However, because a high concentration of sample is required for NMR analysis, the unpurified supernatant (crude enzyme) after cell disruption was used for efficient production of lipid mediators.

### Optimal conditions for catalysis and thermal stability

**Figure 1:**
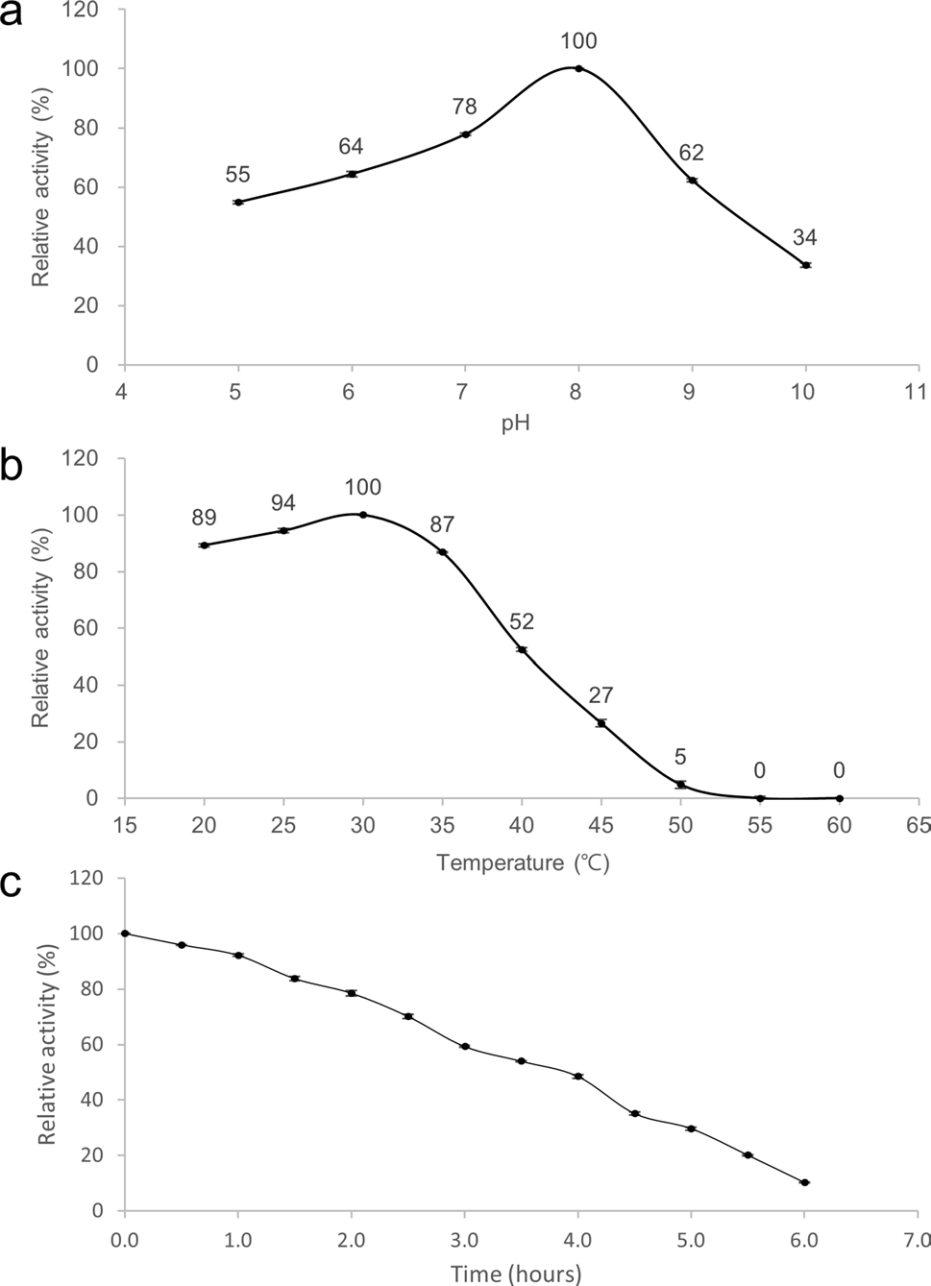
Enzymatic properties of Osc-LOX. (**a**) Effect of pH on the activity. The optimum pH was determined over a range of pH values from 5.0 to 10.0 using 50 mM 2-(*N*-morpholino) ethanesulfonic acid (MES) buffer (pH 5.0), 50 mM sodium phosphate buffer (pH 6.0–7.0), 50 mM Tris–HCl buffer (pH 8.0) and 50 mM sodium tetraborate buffer (pH 9.0–10.0) at room temperature. (**b**) Effect of temperature on the activity. The enzyme reaction for optimum temperature was performed by pre-incubating the reaction mixture without substrate at different temperatures from 20 to 60 °C in 50 mM Tris–HCl buffer (pH 8.0) for 30 min. And then activity was measured following incubation with a substrate. (**c**) Thermal inactivation half-life of Osc-LOX. Substrate-free enzyme mixtures (50 mM Tris–HCl buffer, pH 8.0) were pre-incubated at 30-min intervals up to 6 h, and enzyme activity was measured following incubation with a substrate. All reactions were performed using 3 μg mL^−1^ enzyme and 50 μM substrate (LA, 18:2n-6) for 10 min.

The optimal conditions for catalysis were determined by monitoring the catalytic activity of Osc-LOX at different pH values and temperatures. The effects of pH and temperature on the production of hydroxyoctadecadienoic acid (HODE) from LA (C18:2 n-6^Δ9,12^) were determined using spectroscopic methods. The maximum activity of Osc-LOX was defined as 100%, and the relative activity, as a percentage, was plotted against various pH values and temperature. The relative activity of purified Osc-LOX at various pH values was determined in difference buffer systems at room temperature. Osc-LOX showed optimal activity at pH 8.0 (Fig. 1a). Although it has been reported that some LOXs exhibit optimal activity at an acidic pH, most LOXs have optimal activity at a neutral or alkaline pH^35^. The optimum temperature and thermal stability of Osc-LOX was measured after incubation for 30 min in 50 mM Tris–HCl buffer (pH 8.0) at temperatures between 20 and 60 °C at 5 °C intervals. The optimum temperature for the enzymatic reaction of Osc-LOX was 30 °C, and the enzyme appeared to be stable from 20 to 35 °C with no aggregation (Fig. 1b). However, Osc-LOX aggregated and its activity drastically decreased at temperatures above 40 °C; at temperatures above 55 °C, its activity was completely lost. Osc-LOX might exhibit relative thermal stability at temperatures below 35 °C, but heat denaturation occurs when the temperature rises above 40 °C. The optimum temperature for Osc-LOX was similar to that of soybean LOX, which is maximally active at 30 °C^36^. However, the optimal temperature for Osc-LOX was higher than that for other LOXs, such as human 12-LOX^37^ and *Agrocybe aegerita* LOX4^38^, which show optimal activity at 25 °C. The half-life of the enzyme activity of Osc-LOX was determined by measuring the residual activity by taking the enzyme every 30 min while incubating at 30 °C for 360 min. As a result, Osc-LOX had a half-life of 240 min (Fig. 1c). The time it took for the enzyme activity of Osc-LOX to be decreased in half at the temperature of optimum activity was 1.7 times slower than that of sesame LOX^39^.

### Specific activities and kinetics against PUFAs

The specific activity and kinetics of fatty acid oxyfunctionalization by Osc-LOX were analyzed using highly purified enzyme and the substrates, AA, EPA and DHA, under optimal enzymatic conditions. The Michaelis-Meten plots of each substrates are shown in Supplementary Fig. S5. Michaelis–Menten constants (*K*_m_), turnover numbers (*k*_cat_), catalytic efficiencies (*k*_cat_/*K*_m_), and specific activities of Osc-LOX for different substrates are shown in Table 1. The catalytic efficiency (*k*_cat_/*K*_m_) of Osc-LOX followed the rank order AA (20:4 n-6^Δ5,8,11,14^) > EPA (20:5 n-3^Δ5,8,11,14,17^) > DHA (22:6 n-3^Δ4,7,10,13,16,19^). The specific activity of Osc-LOX towards fatty acids was highest for AA, and followed the rank order AA > EPA > DHA. These results indicate that Osc-LOX prefers arachidonic acid as a substrate and is an ARA 15-LOX. The specific activity of Osc-LOX was 4.5 mmol min^−1^ mg^−1^, which is notably higher than that of other bacterial LOXs. For instance, the specific activity of Osc-LOX towards AA was 193.1-^40^ and 7.4-fold^41^ higher than that of *B. thailandensis* and *M. xanthus* LOXs, respectively.

**Table 1:** Specific activity and kinetic parameters of Osc-LOX.

Substrate	Specific activity^a^ (μmol min^−1^ mg^−1^)	*K*_*m*_ (μM)^a^	*k*_*cat*_ (min^−1^)^a^	*k*_*cat*_*/K*_*m*_ (min^−1^ μM^−1^)^a^
Arachidonic acid (C20)	4486.6 ± 45.3	68.9 ± 3.5	1178.2 ± 16.6	17.1 ± 0.6
Eicosapentaenoic acid (C20)	4941.7 ± 35.2	67.0 ± 3.6	936.2 ± 27.1	14.0 ± 0.4
Docosahexaenoic acid (C22)	5885.2 ± 61.9	63.9 ± 4.8	834.5 ± 20.9	13.1 ± 0.7

^a^Mean values and standard deviations were calculated from three independent experiments.

### Three products generated from DHA by Osc-LOX

**Figure 2:**
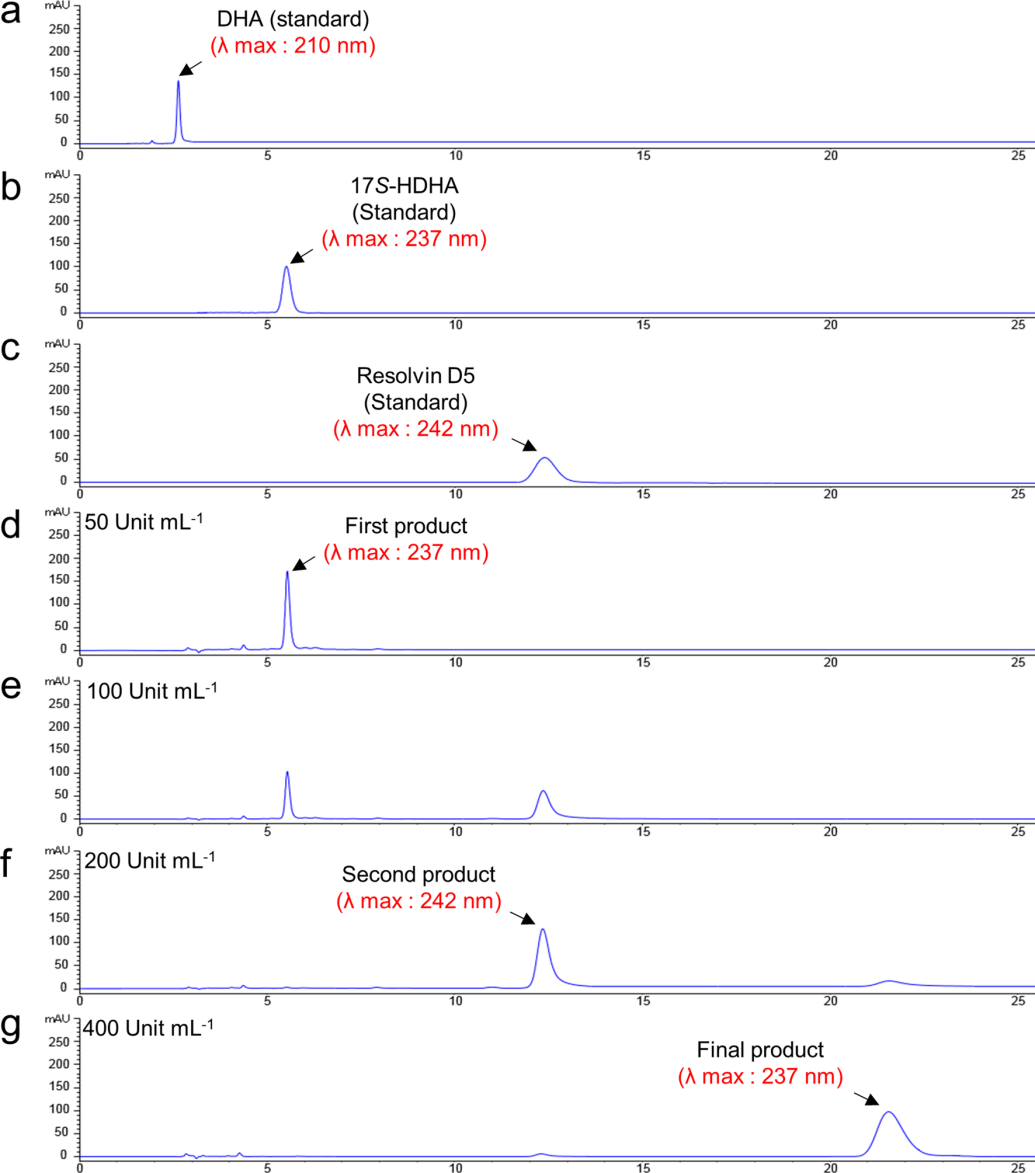
NP-HPLC analysis of three products generated from DHA by Osc-LOX enzymatic catalysis. The reactions were carried out using 50 mM Tris–HCl buffer (pH 8.0), varying amounts of enzyme 50–400 Units mL^−1^, and substrate 50 μM for 30 min at 30 °C. 25 mM sodium borohydride was used for deoxygenation of hydro peroxide products after enzyme reaction. (**a**) DHA standard eluted at 2.65 min. (**b**) 17*S*-HDHA standard eluted at 5.57 min. (**c**) Resolvin D5 standard eluted at 12.3 min. (**d**) First product (17*S*-HDHA) was generated from DHA by Osc-LOX concentration of 50 Unit mL^−1^. (**e**) Second product (RvD5) was generated from 17S-HDHA by Osc-LOX concentration of 100 Unit mL^−1^ but not completely converted. (**f**) When using Osc-LOX concentration of 200 Unit mL^−1^, 17*S*-HDHA was completely converted into RvD5. (**g**) Final product was generated from RvD5 by Osc-LOX concentration of 400 Unit mL^−1^.

In the present study, we focused on the analysis of metabolites converted from DHA (Fig. 2a) by Osc-LOX because the reaction generated previously unknown products. The catalyzed products were identified by NP-, and CP-HPLC analysis.

Three different products were obtained depending on the concentration of Osc-LOX used (50–400 Unit mL^−1^). In NP-HPLC, the first product converted at an Osc-LOX concentration of 50 Unit mL^−1^ eluted at 5.57 min, which matched the retention time of the 17*S*-HDHA standard (17*S*-hydroxy-4*Z*,7*Z*,10*Z*,13*Z*,15*E*,19*Z*-docosahexaenoic acid) at a λ_max_ of 237 nm (Fig. 2b,d). At 50 Unit mL^−1^, Osc-LOX completely consumed DHA, but the concentration of the converted product was 45 μM (conversion rate from DHA, 95%).

Additional conversion products of DHA catalyzed by Osc-LOX at concentrations of 100 to 200 Unit mL^−1^ were detected at 12.3 min at a λ_max_ of 242 nm identical to the resolvin D5 (RvD5; 7*S*,17*S*-dihydroxy-4*Z*,8*E*,10*Z*,13*Z*,15*E*,19*Z*-docosahexaenoic acid) standard (Fig. 2c,e). The first product was converted into the second product at a concentration of 100 Unit mL^−1^, but full conversion was achieved using an Osc-LOX concentration of 200 Unit mL^−1^, which yielded 38 μM RvD5, a conversion rate of 84.5% from the first product (Fig. 2f). The concentrations of 17*S*-HDHA and RvD5 were calculated using commercially available standards.

Surprisingly, at an Osc-LOX concentration of 400 Unit mL^−1^, we obtained a remarkable peak at 21.56 min at a λ_max_ of 237 nm; the final product was obtained at 32 μM, corresponding to a conversion rate of 82.2% from the second product (Fig. 2g). The concentration of the final product was determined by reference to a calibration curve generated using purified products prepared by preparative HPLC.

Our assumption is that this latter peak is a new type of lipid mediator, as supported by several lines of evidence. First, this peak did not overlap with the retention time of any of the resolvin D series standards (RvD1-RvD5 and protectin DX; standards spectra not shown). Second, each product was further converted in a manner that depended on the enzyme concentration, indicating that the final product was generated from the second product (RvD5). Because LOX is known to be an irreversible enzyme^42^, higher concentrations are needed to catalyze monohydroperoxy fatty acids to dihydroperoxy fatty acids. Thus, LOX might lose its activity before being able to catalyze the next oxygenation. If the final product was converted from the second product (RvD5), one double bond must be eliminated because the final product had singlet peak at a λ_max_ of 237 nm; its spectrum indicated that it has one conjugated diene. Lipid mediators have unique absorption spectra reflecting their conjugated double bonds.

In addition, the stereo-configuration of products generated from DHA by Osc-LOX were analyzed by CP-HPLC (OD-H column) and compared with 17*S*-HDHA, 17*R*-HDHA and RvD5 standards. The retention times of the first product was 9.22 min, which is confirmed as 17*S*-HDHA. And the retention times of the second product was 38 min, which is identical to RvD5 standard. Although the second product did not be compared with the stereoisomer, 7*R*,17*S*-diHDHA, through CP-HPLC analysis, it has been reported that RvD5 and its stereoisomer have different retention times^43^. The final product stayed on the OD-H column for 82 min and its peak did not overlap those for any of the standards, as expected (Supplementary Fig. S6). These results indicate that the first and second products of the catalysis of DHA by Osc-LOX also matched the stereo-configuration as well as the regio-configuration of 17*S*-HDHA and 7*S*,17*S*-diHDHA (RvD5), further supporting the possibility that the final product may be a new type of lipid mediator.

### Structure identification of conversion products of DHA by Osc-LOX

To identify conversion products, we first confirmed its molecular mass by performing LC–MS analysis in negative mode. The first product detected had an *m/z* of 343.2 [M−H^−^], corresponding to the molecular mass of 17S-HDHA (344.5 Da) (Supplementary Fig. S7a), and the second product had an *m/z* of 359.2 [M−H^−^], corresponding to the molecular mass of RvD5 (360.5 Da) (Supplementary Fig. S7b). The final product had an *m/z* of 375.2 [M−H^−^] (Supplementary Fig. S7c), which was identical to tri-hydroxy lipid mediators with six double bonds, such as RvD1 and RvD2, but HPLC analysis showed that it did not match previously known resolvin D series compounds. The final product was further analyzed by LC–MS/MS. Fragment peaks were detected at *m/z* values of 113 (235.1, 141) and 265.1 by cleavage between C6–C7, C14–C15, and C15–C16 positions (Supplementary Fig. S8). This MS/MS analysis of the final product suggests that, because the molecular weight of the fragment containing the methyl group formed by cleavage between C14 and C15 was detected as an *m/z* of 141, the final product must reflect removal of a double bond with formation of an epoxide group. Thus, the final product should have two hydroxyl groups and one epoxide group, based on the total molecular weight or molecular weight of fragments.

The structural configurations of the three products generated from DHA by Osc-LOX were more accurately determined by 1D and 2D NMR spectroscopy. The first and second products were assigned using ^1^H, ^13^C, COSY, TOCSY, Edited-HSQC, and HMBC spectra. The first product was identified as 17*S*-hydroxy-4*Z*,7*Z*,10*Z*,13*Z*,15*E*,19*Z*-docosahexaenoic acid (17*S*-HDHA). Chemical shifts of the C17-OH hydroxyl group in the first product were recorded at 4.12 ppm (^1^H) and 73.99 ppm (^13^C) (Supplementary Figs. S9 and S10, and Table S3a). The second product was identified as 7*S*,17*S*-dihydroxy-4*Z*,8*E*,10*Z*,13*Z*,15*E*,19*Z*-docosahexaenoic acid (RvD5). The two hydroxyl groups, C7-OH and C17-OH, of the second product were detected as overlapping peaks at 4.13 ppm (^1^H) and 73.93–73.96 ppm (^13^C), respectively (Supplementary Figs. S11 and S12, and Table S3b). Two of the three products were confirmed to be identical to previously reported chemical structures of 17*S*-HDHA and RvD5 (7*S*,17*S*-diHDHA), identified by Dobson^44^. The specific rotation values of two products were 17*S*-HDHA [α]_D_ + 3.6° (*c* 0.1, EtOH) and 7*S*,17*S*-diHDHA [α]_D_ + 4.9° (*c* 0.1, EtOH).

The final product was assigned using COSY, TOCSY, NOESY, Edited-HSQC, and HMBC spectra. The structure of the novel lipid mediator was identified as 7*S*,15*R*-dihydroxy-16*S*,17*S*-epoxydocosa-4*Z*,8*E*,10*Z*,13*Z*,19*Z*-pentaenoic acid, which has two hydroxyl groups introduced at C7 and C15, with an epoxide group formed across C16 and C17. Chemical shifts corresponding to C7-OH, C15-OH, and C16-C17 (epoxide ring) were recorded as follows: C7-OH, 4.15 ppm (^1^H) and 73.71 ppm (^13^C); C15-OH, 4.23 ppm (^1^H) and 69.97 ppm (^13^C); C16-epoxide, 2.79 ppm (^1^H) and 62.79 ppm (^13^C); C17-epoxide, 2.9 ppm (^1^H) and 57.42 ppm (^13^C) (Supplementary Figs. S13–S15, and Table S3c). C7-H and C17-H were identified as the *S*-form because the precursor (RvD5) has a 7*S*, 17*S* stereo-configuration. Epoxide ring was determined to be the *trans* geometry based on the coupling constants of J16 and J17, which are 2.4 Hz and 2.2 Hz, respectively. Thus, C16-H was determined to be the *R*-form because C17-H has an *S*-form configuration. In addition, C15-H was identified as *R*-form because a strong NOE correlation signal was monitored between C17-H confirmed as *S*-form and C15-H in NOESY NMR (Supplementary Fig. S16). Although C15 is closer to C16 than C17, NOE signal was detected very weakly between C15-H and C16-H. This result indicates that a hydrogen atom of C15 has an opposite direction to a hydrogen atom of C16 hydrogen having an *S*-form configuration, and has a similar direction as a hydrogen atom of C17. The specific rotation value of 7*S*,15*R*-dihydroxy-16*S*,17*S*-epoxy-docosapentaenoic acid was measured as [α]_D_ + 2.1° (*c* 0.1, EtOH).

Ultimately, final product was confirmed as a new type of lipid mediator because it does not match any molecules registered in chemical databases. Lipoxygenases classified as ARA15LOX type are known to convert intermediate 17*S*-HDHA into 7*S*,17*S*-dihydroxy-docosahexaenoic acid as RvD5 and 10*S*,17*S*-dihydroxy-docosahexaenoic acid as PDX^45^. Here, our study is the first to report the production of new structure lipid mediator via RvD5 converted from 17*S*-HDHA.

### Two products converted from 7*S*,15*R*-dihydroxy-16*S*,17*S*-epoxy-docosapentaenoic acid

**Figure 3:**
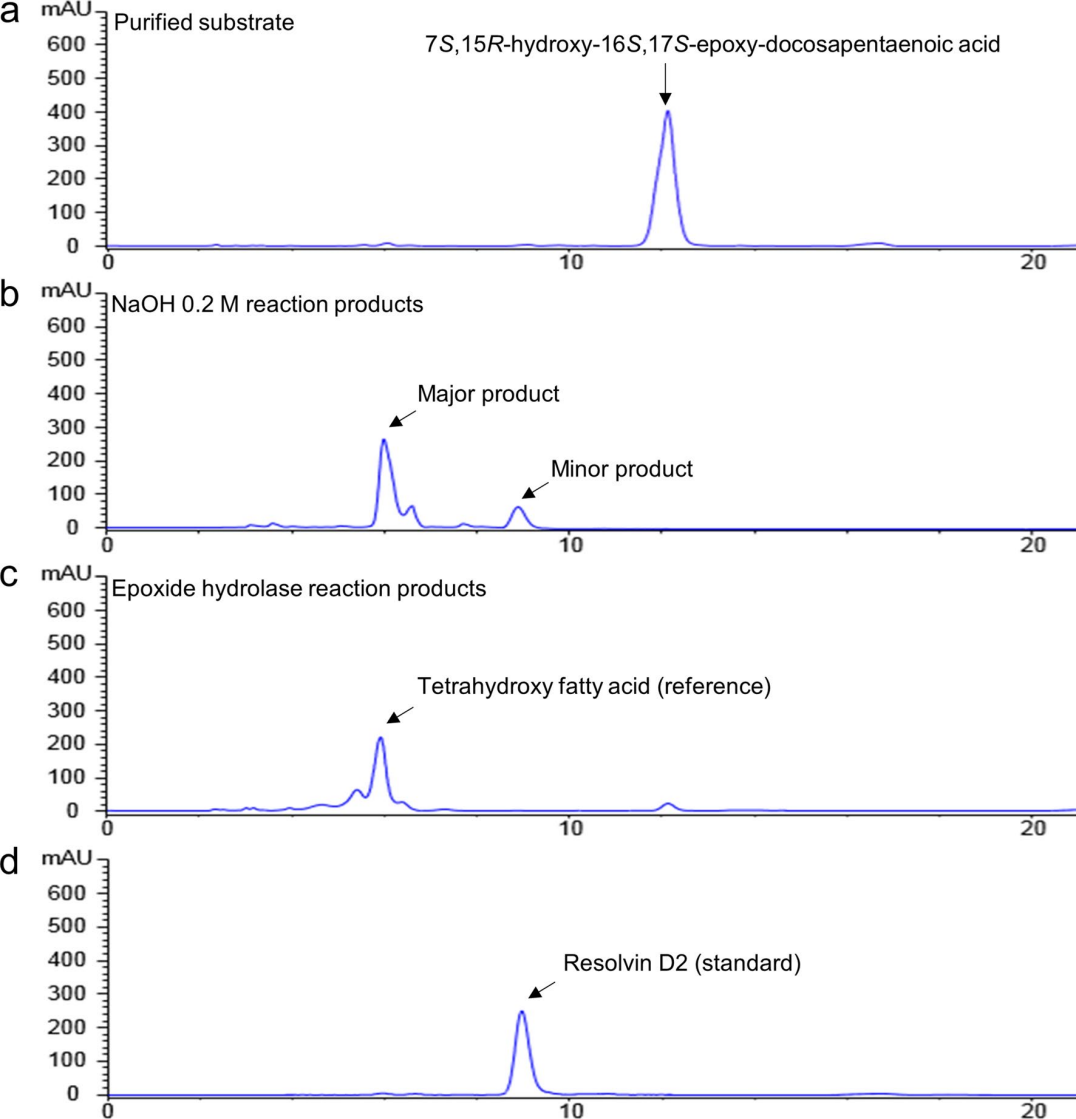
RP-HPLC analysis of epoxide ring-opened products by sodium hydroxide (NaOH). (**a**) Purified 7*S*,15*R*-dihydroxy-16*S*,17*S*-epoxy-docosapentaenoic acid as a precursor. (**b**) Two products were generated by NaOH: main product was 7*S*,15*R*,16*S*,17*S*-tetrahydroxy docosapentaenoic acid (conversion rate of 90% or less) and minor product was RvD2 (conversion rate of 10%). (**c**) Tetrahydroxy fatty acid was produced by epoxide hydrolase (EH; commercially available) to use as a reference. (**d**) RvD2 standard. The epoxide ring-opening reaction was performed with NaOH at 30 °C overnight.

We performed a hydrolysis reaction for epoxide ring-opening on purified 7*S*,15*R*-dihydroxy-16*S*,17*S*-epoxy-docosapentaenoic acid (Fig. 3a) using sodium hydroxide (NaOH). The converted products were confirmed using RP-HPLC (C18) and RP-chiral HPLC. RP-HPLC analysis showed that 7*S*,15*R*-dihydroxy-16*S*,17*S*-epoxy-docosapentaenoic acid was completely consumed and converted into two products: a main product with a conversion rate of 90% or less (~ 90 μg), and a minor product accounting for 10% (10 μg). The concentration of the minor product was determined by reference to a calibration curve prepared using a commercially available resolvin D2 (RvD2, 7*S*,16*R*,17*S*-trihydroxy docosahexaenoic acid) standard. However, the concentration of the major peak was estimated by subtracting the amount of the quantified minor peak from the concentration of the starting material because a standard curve could not be generated for the major peak owing to difficulties in purification. The retention times of the major and minor products were 6 and 8.8 min, respectively, values that were identical to those of the standards (Fig. 3b). These standards correspond to the conversion product of the EH reaction and commercially available RvD2 (Fig. 3c,d). In addition, the chirality of a minor product was identified by a RP-chiral HPLC (IB column) comparing with RvD2 standards. The retention times of the minor product was 6.78 min, which are identical to RvD2 standard (Supplementary Fig. S17). EHs have been known to catalyze the conversion of an epoxide ring into diol in vivo. However, the base-catalyzed epoxide ring-opening method using NaOH could be more cost-effective, and simple than EH enzyme reaction.

### Structure identification of two products produced by hydrolysis

A MS/MS analysis showed that the total molecular mass of the major product was represented by a peak at an *m/z* of 393.2 [M−H^−^]. This result indicates that two hydroxyl groups were generated by epoxide ring-opening, resulting in an 18 Da increased in total molecular weight of the major product compared to 7*S*,15*R*-dihydroxy-16*S*,17*S*-epoxy-docosapentaenoic acid (376.22 Da) owing to addition of two hydrogens (2 Da) and one oxygen (16 Da). The fragments showed peaks at *m/z* values of 113.05, (235.1, 159.1) and 265.1. The two peaks at *m/z* 159.1 and 235.1 resulted from cleavage between C14 and C15 (Supplementary Fig. S18a). These results provide additional supporting evidence that the two hydroxyl groups form by epoxide ring-opening because the molecular weight of the fragment containing the methyl group formed by cleavage between C14 and C15 was detected as an *m/z* of 159.1. Thus, the major product contains four hydroxyl groups. The minor product obtained by hydrolysis of the epoxide ring was identified as RvD2 using MS/MS analysis. The total molecular mass of the minor product and the RvD2 standard (376.5 Da) were identical, with an *m/z* of 375.2 [M−H^−^]. Fragmented peaks recorded from the minor product yielded precisely the same pattern as the RvD2 standard, with *m/z* values of 69.02, (113.05, 233.1) and 247.1 (Supplementary Fig. S18b,c). The specific rotation value of 7*S*,16*R*,17*S*-trihydroxy docosahexaenoic acid was [α]_D_ − 14.8° (*c* 0.05, EtOH).

The chemical structure of the major product converted by epoxide ring-opening was precisely identified by NMR analysis using COSY, TOCSY, NOESY, Edited-HSQC, and HMBC methods. The structure of the tetrahydroxy fatty acid was identified as 7*S*,15*R*,16*S*,17*S*-tetrahydroxy-4*Z*,8*E*,10Z,13*Z*,19*Z*-pentaenoic acid. Chemical shifts corresponding to C7-OH, C15-OH, C16-OH, and C17-OH were detected as follows: C7-OH, 4.14 ppm (^13^H) and 73.91 ppm (^13^C); C15-OH, 4.69 ppm (^13^H) and 68.51 ppm (^13^C); C16-OH, 3.29 ppm (^13^H) and 79.09 ppm (^13^C); C-17-OH, 3.64 ppm (^13^H) and 73.68 ppm (^13^C). (Supplementary Figs. S19–S21, and Table S3d). These results show that chemical shifts of the newly formed two hydroxyl groups at C16 and C17 by ring opening were significantly changed compared with 7*S*,15*R*-dihydroxy-16*S*,17*S*-epoxy-docosapentaenoic acid (Supplementary Fig. S22). Stereochemical configuration of C7-OH and C15-OH was determined as the *S*- and *R*-form, respectively, because the precursor was identified as 7*S*- and 15*R*-form. In the NOESY spectrum, NOE correlation signal was detected between C15-H and C16-H, and C16-H and C17-H, respectively (Supplementary Fig. S23). However, unlike the NOESY NMR result of 7*S*,15*R*-dihydroxy-16*S*,17*S*-epoxy-docosapentaenoic acid, NOE signal was not detected between C15 and C17. This result showed that the chirality of carbon 16 was reversed when the epoxide ring was opened. The specific rotation value of 7*S*,15*R*,16*S*,17*S*-tetrahydroxy-pentaenoic acid was measured as [α]_D_ − 6.4° (*c* 0.1, EtOH).

7*S*,15*R*,16*S*,17*S*-tetrahydroxy-4*Z*,8*E*,10*Z*,13*Z*,19*Z*-pentaenoic acid was also identified as a new type of lipid mediator because it does not match any molecules registered in chemical databases. We found we could accomplish two things at once because 7*S*,15*R*-dihydroxy-16*S*,17*S*-epoxy-docosapentaenoic acid was further converted to 7*S*,15*R*,16*S*,17*S*-tetrahydroxy docosapentaenoic acid and RvD2 by base-promoted epoxide ring-opening. This finding is interesting because, although an additional chemical reaction is required, it bypasses the difficulty and high cost of preparing an additional enzyme (EH) for converting epoxide into diol; moreover, RvD2 did not be converted by EH. To date, RvD2 has been produced only by total synthesis methods, with no reports of the efficient production of RvD2 by an in vitro enzymatic reaction. However, total synthesis methods have some limitations, including low yield, toxicity of chemicals, and the time-consuming 20-step process required^12^. The combinatorial approach using both enzymatic and chemical reactions described here could be a very attractive option for overcoming difficulties in the production of RvD2.

### 3D structure and molecular docking of the Osc-LOX

**Figure 4:**
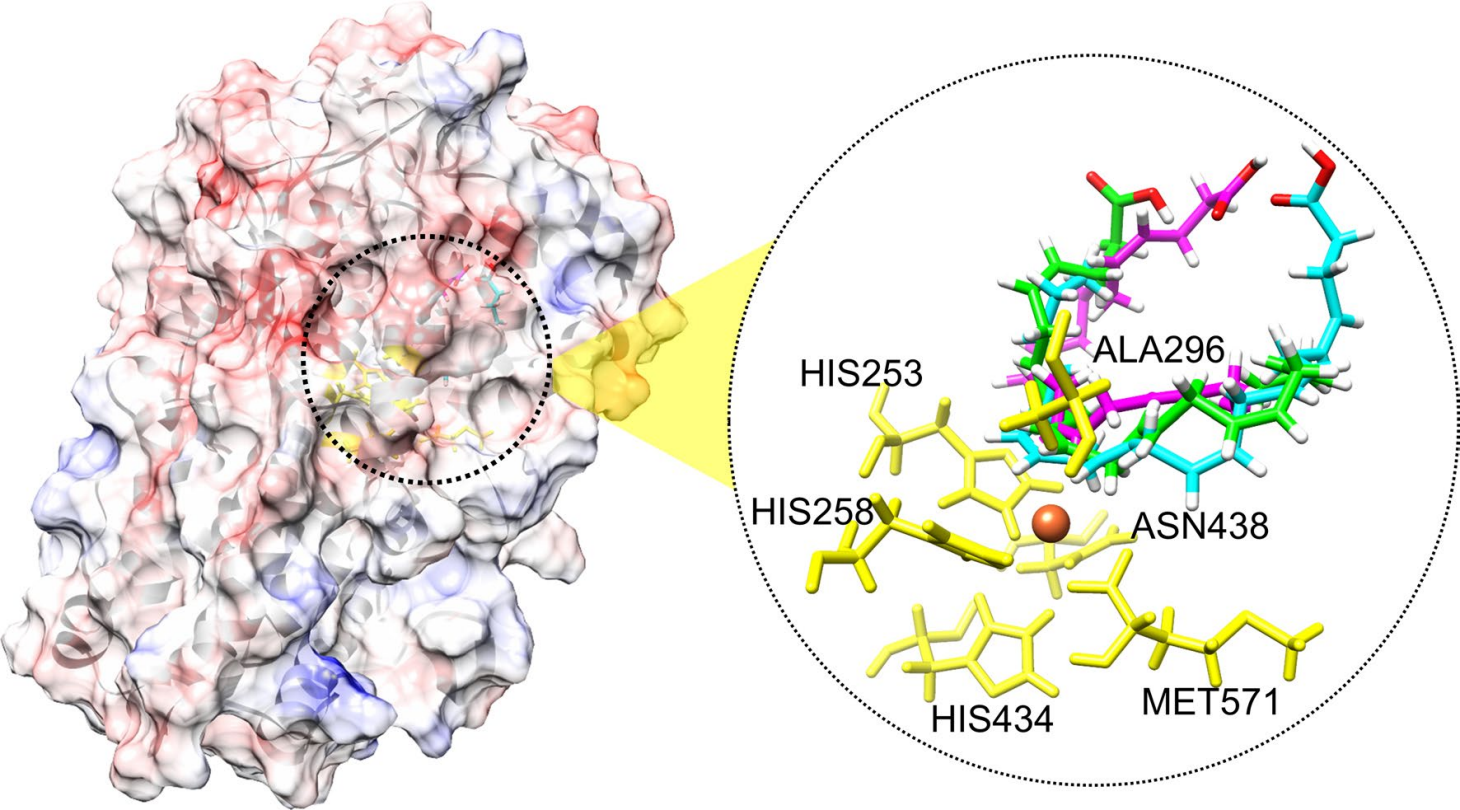
Complex models of lipid mediators and Osc-LOX. Surface model of Osc-LOX was represented with the electrostatic surface potential colored as blue (positively charged region) and red (negative charged region) with 10 scaled energy kcal/(mole*e) unit. The substrate in the catalytic site of Osc-LOX were shown with stick presentation method, colored by cyan (binding pose for 17*S*-HDHA), green (binding pose for RvD5), and magenta (binding pose for 7*S*,15*R*-dihydroxy-16S,17*S*-epoxy-docosapentaenoic acid), respectively. Six conserved residues in Osc-LOX were colored as yellow including 253His, 258His, 296Ala, 434H, 438N, and 571Met. And Fe ion was represented as orange sphere in catalytic site. UCSF Chimera 1.11.2 (https://www.cgl.ucsf.edu/chimera/).

**Figure 5:**
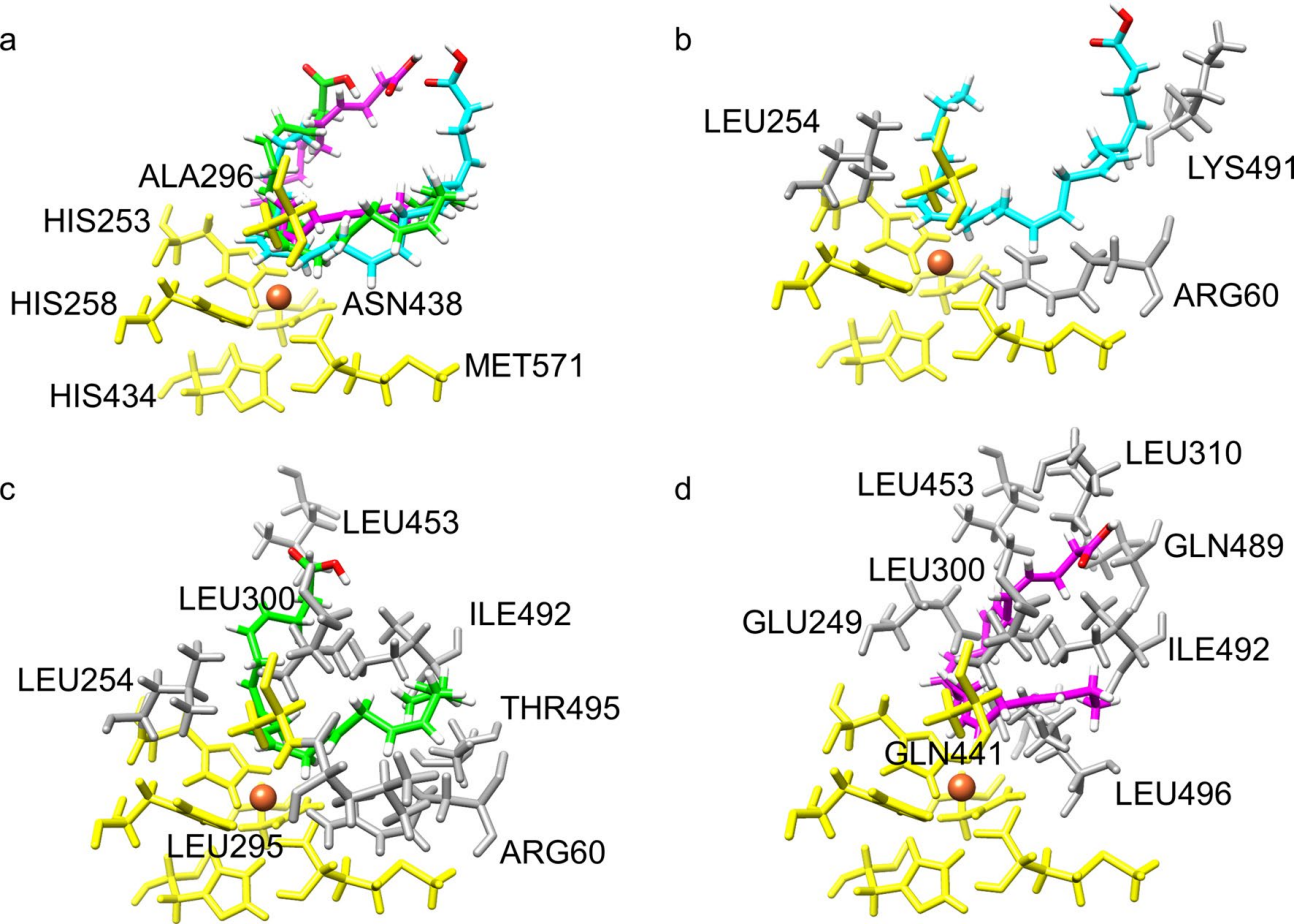
Binding poses of substrate in catalytic site of Osc-LOX. (**a**) Superposed image of potential binding poses of substrate in catalytic site of Osc-LOX. (**b**) Binding pose for 17*S*-HDHA in catalytic site of Osc-LOX. (**c**) Binding pose for RvD5 in catalytic site of Osc-LOX. (**d**) Binding pose for 7*S*,15*R*-dihydroxy-16*S*,17*S*-epoxy-docosapentaenoic acid in catalytic site of Osc-LOX. Metal ion was represented by orange sphere. The conserved amino acids in catalytic site were indicated by yellow sticks, and amino acids of Osc-LOX within 2 Å from each substrate were presented by dark gray sticks. UCSF Chimera 1.11.2 (https://www.cgl.ucsf.edu/chimera/).

A 3D structural analysis of the catalytic site of the Osc-LOX protein also showed that multiple products could be produced from the DHA substrate. Because the 3D structure of the Osc-LOX protein has not yet been elucidated, the binding pose of the DHA substrate at the catalytic site was predicted using a 3D structure generated by homology modeling (Figs. 4 and 5). In the predicted structure of Osc-LOX, like other LOX proteins, conserved amino acid residues (His253, His258, His434, Asn438, and Met571) are known to bind a metal ion, and Ala296 is close to the catalytic site and affects the binding pose of the substrate^46^. Ala296 is known to be an important residue that regulates regioselectivity during catalysis of other LOX proteins, an interaction that is explained by the Coffa theory.

In addition, using AutoDock Vina, we were able to calculate the binding energy for each binding pose (B, 8.2020 kcal/mol; C, 7.6950 kcal/mol; and D, 6.7720 kcal/mol), which predicts that substrate-enzyme complexes could be readily formed at higher binding energies (Fig. 5). These results indicate that low concentrations of Osc-LOX produce an intermediate complex predominantly in the form of the energy-favorable 17*S*-hydroperoxy-DHA. On the other hand, it suggests that an intermediate complex that produces 7*S*,17*S*-dihydroperoxy-DHA and 7*S*,15*R*-dihydroperoxy-16*S*,17*S*-epoxy-DPA could be formed as the concentration of Osc-LOX increases.

### Conversion pathway of DHA into lipid mediators by combinatorial catalysis

**Figure 6:**
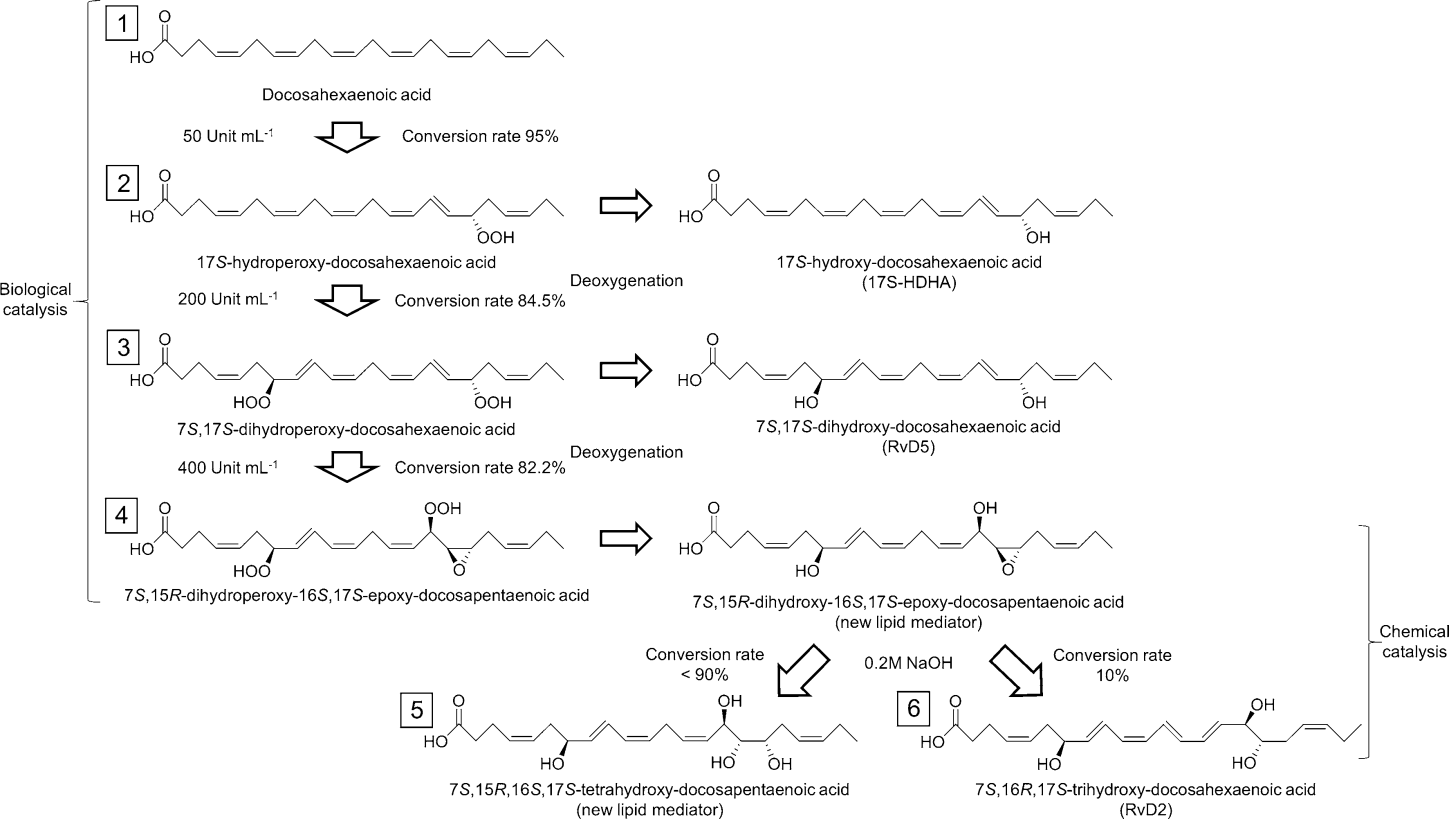
Conversion pathways of DHA into lipid mediators by collaboration with biological and chemical reactions. Three products were converted from DHA by Osc-LOX, in which the conversion proceeds sequentially in a concentration-dependent manner of the enzyme. Osc-LOX converted DHA (1) into 17*S*-hydroperoxy-docosahexaenoic acid (2), 17*S*-hydroperoxy-docosahexaenoic acid into 7*S*,17*S*-dihydroperoxy-docosahexaenoic acid (3), and 7*S*,17*S*-dihydroperoxy-docosahexaenoic acid into 7*S*,15*R*-dihydroperoxy-16*S,*17*S*-epoxy-docosapenta-enoic acid as a precursor, respectively. And then, 17*S*-HDHA, RvD5, and 7*S*,15*R*-dihydroxy-16*S*,17*S*-epoxy-docosapentaenoic acid were converted by deoxygenation using sodium borohydride from each corresponded precursor. (4). Two products were generated by sodium hydroxide (NaOH): main product was 7*S*,15*R*,16*S*,17*S*-tetrahydroxy-docosapentaenoic acid (5) and minor product was RvD2 (6).

Five types of bioactive hydroxyl fatty acids have been successfully converted from DHA used as start material via collaboration including enzymatic reaction and base promoted epoxide ring-opening. Three lipid mediators were produced by Osc-LOX. The conversion rate and purity of 17S-hydroxy-DHA from DHA were 95%, respectively. RvD5 (7*S*,17*S*-dihydroxy-DHA) was generated from 17*S*-hydroperoxy-DHA with 84.5% conversion rate and its purity was 76%. The yield of 7*S*,15*R*-dihydroxy-16*S,*17*S*-epoxy-DPA produced from 7*S*,17*S*-dihydroperoxy-DHA was 82.2% and the purity was 64%. The yield and purity of two products generated from 7*S*,15*R*-dihydroxy-16*S,*17*S*-epoxy-DPA by NaOH were followed: 7*S*,15*R*,16*S*,17*S*-tetrahydroxy-DPA (< 90% and < 90%); 7S,16R,17S-trihydroxy-DHA (10% and 10%). The combinatorial synthetic pathway is depicted in Fig. 6, and all of the lipid mediators produced in this study are presented in Supplementary Table S4.

## Conclusion

Discovering new lipoxygenases that have not been functionally characterized could be a powerful strategy for the production of various lipid mediators that could have high value-added. Thus, we identified a unique lipoxygenase, named Osc-LOX, derived from cyanobacteria. The specific activity against AA was 4.5 mmol min^−1^ mg^−1^, which is significantly higher compared to mammalian or other bacterial LOXs. DHA was converted into three lipid mediators, 17*S*-HDHA, RvD5, and a new type of 7*S*,15*R*-dihydroxy-16*S*,17*S*-epoxydocosa-4*Z*,8*E*,10*Z*,13*Z*,19*Z*-pentaenoic acid by enzymatic catalysis. In addition, another new type of 7*S*,15*R*,16*S*,17*S*-tetrahydroxy-4*Z*,8*E*,10*Z*,13*Z*,19*Z*-pentaenoic acid and RvD2 were converted by base-promoted epoxide ring-opening reaction from 7*S*,15*R*-dihydroxy-16*S*,17*S*-epoxy-DPA. This application of such a cost-effective and eco-friendly production system could be essential for the commercialization of lipid mediators in the cosmetic and pharmaceutical industries.

## Supplementary information

Supplementary Information.
