# Supplementary material for: Synthesis of two new lipid mediators from docosahexaenoic acid by combinatorial catalysis involving enzymatic and chemical reaction
Source: Sci Rep. 2020 Nov 2;10:18849. doi: 10.1038/s41598-020-76005-5 (PMC7606508; doi:10.1038/s41598-020-76005-5)
Supplement: Supplementary file 1 — Supplementary Information. [file 41598_2020_76005_MOESM1_ESM.pdf]

Supplementary Information for:

Synthesis of two new lipid mediators from docosahexaenoic acid by combinatorial catalysis involving enzymatic and chemical reaction

Jong-Jae Yi,[1,2] Sun-Yeon Heo,[1] Jung-Hyun Ju,[1] Baek-Rock Oh,[1] Woo Sung Son,[2,*] Jeong-Woo Seo[1,*]

[1]Microbial Biotechnology Research Center, Korea Research Institute of Bioscience and Biotechnology (KRIBB), Jeongeup-Si, 56212, Republic of Korea

[2]Department of Pharmacy, College of Pharmacy and Institute of Pharmaceutical Sciences, CHA University, Pocheon-Si, Gyeonggi-do, 11160, Republic of Korea

ATGGTAGACAATATGAAACCGTCTCTTCCTCAAGACGACCCGAACCAAGAACAGCGCAAGGACTCCTTGAATCGCCAGCAGCAAG
CTTATCAGTTTGACTATGAGAGTTTATCACCATTGGCATTATTGAAAAACGTGCCCGCAGTTGAGAACTTTTCGTCAAAGTATATTGG
GGAAAGAATATTAGCAACATCGGAACTTCCAGCAAATATGCTGGCAGCGGATTCTAGAACTTTTCTGGATCCTCTCGATGAACTCCA
AGATTATGAGGATTTCTTTACTCTGCTGCCGCTGCCTGCTGTTGCTAAAATTTACCAAACCGATCGCTCTTTCGCAGAACAGCGCCT
GTCTGGAGCAAACCCGATGGTGCTTCGTCTGTTAGATGCCGGCGATCCGCGGGCGCAAACACTGGCACAAATTTCCAGCTTTCAT
CCATTATTCGATCTGGGCCAAGAGTTGCAGCAAAAAAACATTTATGTGGCCGATTACACGGGTACTGACGAACACTATCGCGCGCC
GTCAAAGATAGGAGGCGGGAGCTATGAAAAAGGCAGAAAATTCCTGCCGAAACCGCGGGCTTTTTTCGCATGGCGGTGGACGGG
GATTCGCGATCGCGGTGAAATGACACCAATTGCCATACAACTAGATCCCACGCCAGATAGCCATGTCTACACCCCATTCGACCCTC
CTGTGGATTGGCTGTTTGCGAAACTCTGTGTGCAAGTAGCAGATGCCAATCACCATGAAATGAGCTCGCATTTAGGTCGTACGCAT
CTGGTGATGGAACCAATTGCGATCGTAACCGCCCGTCAGTTGGCCCAAAATCATCCGCTGAGCCTGTTGCTGAAACCGCACTTTC
GCTTTATGCTTACCAACAACGAGCTGGCACGTTCTTATCTAATCGCTCCCGGTGGGCCCGTCGACGAACTTCTAGGCGGTACTCTT
CCAGAAACAATGGAGATAGCTAGAGAGGCTTGTAGTACCTGGAGTCTCGATGAGTTTGCGTTGCCCGCCGAACTGAAAAATCGTG
GCATGGATGACACAAATCAGCTGCCTCACTATCCGTATCGAGACGATGGACTTCTGCTTTGGGATGCGATAGAGACGTTTGTTTCC
GGCTATCTGAAATTCTTTTATCCGACGGAGATCGCGATCGTACAAGATGTTGAACTGCAGACCTGGGCCCAAGAATTAGCGTCCGA
TAGGGGCGGTAAGGTCAAAGGAATGCCTCCACGCATCAATACCGTTGAACAGTTAATTAAAATCGTGACAACTATAATTTTCACCTG
CGGTCCGCAGCATTCAGCAGTCAACTTTCCACAGTATGAATACATGAGTTTTGCCGCCAATATGCCGTTGGCAGCGTACCGTGATA
TTCCCAAAATTACTGCTTCAGGCAATCTCGAAGTTATAACAGAAAAAGACATCTTACGGCTTTTACCTCCGTACAAACGAGCGGCTG
ACCAGCTGAAAATTCTGTTTACTCTGTCAGCTTATAGGTATGACCGTTTGGGTTACTACGATAAATCTTTTCGTGAACTGTATCGGAT
GAGTTTCGATGAGGTTTTTGCAGGAACCCCGATCCAGCTTTTAGCCCGTCAGTTCCAGCAGAACTTAAATATGGCAGAACAAAAGA
TTGATGCCAACAATCAAAAGCGAGTTATTCCTTACATTGCTCTCAAGCCTTCCTTGGTAATCAATAGCATCAGTATGTAA

Fig. S1 Synthesized sequence of *osc-lox* with E.coli codon optimization.

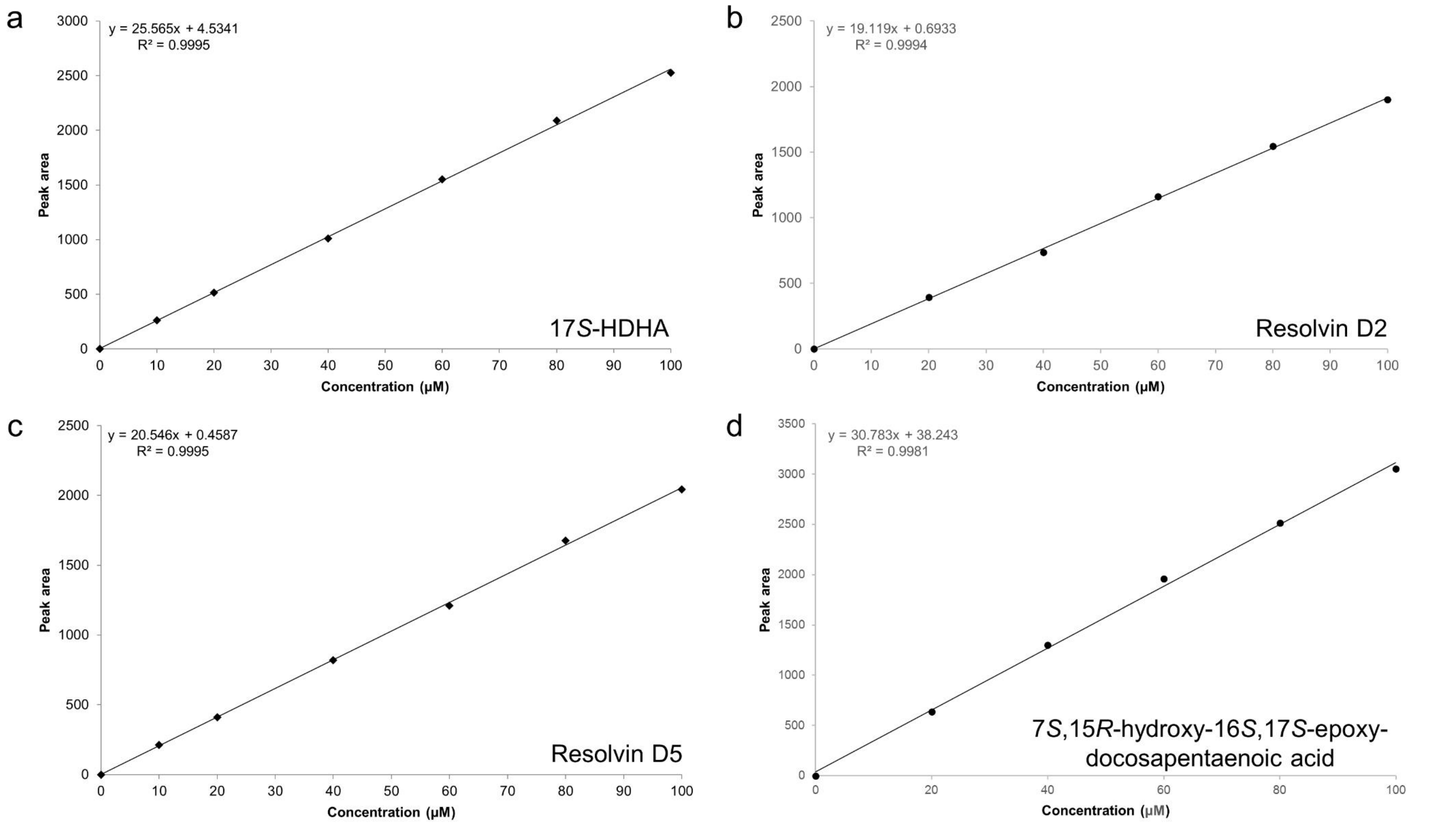

Fig. S2 Standard curve of conversion products of DHA. (a) 17S-HDHA, (b) Resolvin D2, (c) Resolvin D5, and (d) 7S,15R-hydroxy-16S,17S-epoxy-docosapentaenoic acid.

	Metal ligand	Coffa determinant	Metal ligand	
Human A5(S)LOX	368 H L L R T H	410 K A R	551 H A A V N	671 S V A I
Human A15(S)LOX	360 H L L R G H	402 R A R	540 H A S V H	659 S V A I
Mouse A5(S)LOX	368 H L L R T H	410 K A R	554 H A A V N	671 S V A I
Mouse A8(S)LOX	374 H L L H A H	416 L A R	554 H A A V S	674 S V S I
Mouse A15(S)LOX	361 H L L R G H	403 R A R	541 H S S I H	660 S V A I
Rabbit A15(S)LOX	361 H L L R G H	403 R A R	541 H S S I H	660 S V A I
Potato L9(S)LOX	522 H W L N T H	564 S A R	713 H A A V N	858 S V S I
Potato L13(S)LOX	557 H W L R T H	599 L A R	749 H A A V N	896 S I S I
Soybean L9(S)LOX	518 H W L N T H	560 L A R	709 H A A V N	854 S I S I
Soybean L13(S)LOX	499 H W L N T H	541 L A R	690 H A A V N	836 S I S I
Pseudomonas aeruginosa A15(S)LOX	377 H L A Q T H	419 G A A	555 H A A V N	682 S T N I
Cyanothece sp PCC8801 A15(S)LOX	257 H L A R T H	299 L A R	449 H A A V N	566 S T S I
Myxococcus xanthus DK 1622 A12(S)LOX	405 H L G Q C H	446 S A N	614 H A W A N	712 R I N I
Pyropia haitanensis L13(S)LOX	585 H L G M T H	627 L A R	774 H A A V N	896 S I D I
Arabidopsis thaliana L9(S)LOX	519 H W M Q T H	561 L A R	771 H A A V N	856 S V S I
Hordeum vulgare 9(S)LOX	517 H W L N T H	559 L A R	708 H A A V N	859 S I S I
Arabidopsis thaliana L13(S)LOX	554 H W L R T H	596 R A R	746 H A A V N	893 S I S I
Hordeum vulgare L13(S)LOX	587 H W L R T H	629 Q A R	777 H A A V N	933 S I S I
Oscillatoria nigroviridis PCC7112 LOX	253 H L G R T H	295 L A R	434 H S A V N	568 S I S M
Human A12(R)LOX	398 H L L E T H	440 I G R	578 H A A V N	698 S I S I
Mouse A12(R)LOX	398 H L L E S H	440 I G R	578 H A A V N	698 S I S I
Plexaura homomalla A8(R)LOX	408 H L L K T H	450 V G I	594 H H A I N	712 S I H T
Pyricularia oryzae L11(R)LOX	298 H V A Y P H	340 L G E	438 H H V L N	616 Y L S V
Gaeumannomyces avenae L13(R)LOX	290 H V L F - H	331 V G G	478 H H V M N	615 F L S V

Fig. S3 Sequence alignment analysis of the catalytic site of lipoxygenases (LOXs).

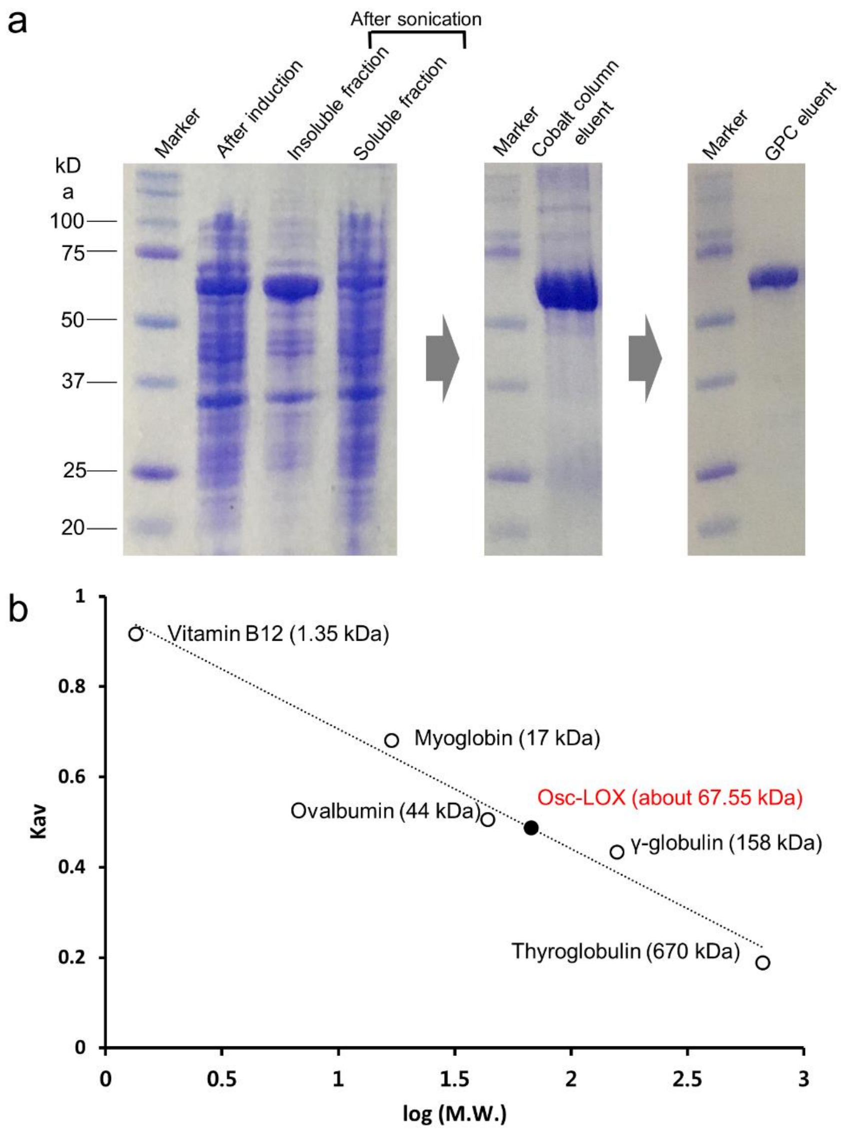

Fig. S4 Purification and molecular weight determination of Osc-LOX. (a) Left panel, expression and solubility of Osc-LOX were confirmed by SDS-PAGE. The molecular weight of Osc-LOX (67.2 kDa) containing a His-tag. Middle panel, Osc-LOX was purified by cobalt affinity chromatography as a first step. Right panel, Osc-LOX was highly purified by size-exclusion chromatography (SEC) resulting in a concentration of 3mg mL^{-1}. (b) The molecular weight and oligomeric state of purified Osc-LOX were determined using SEC on a HiLoad 16/600 Superdex 200 pg. The empty circles indicate the reference proteins. Osc-LOX was eluted at a position corresponding to 67.5 kDa (filled circle) and expressed as a monomer in aqueous solution.

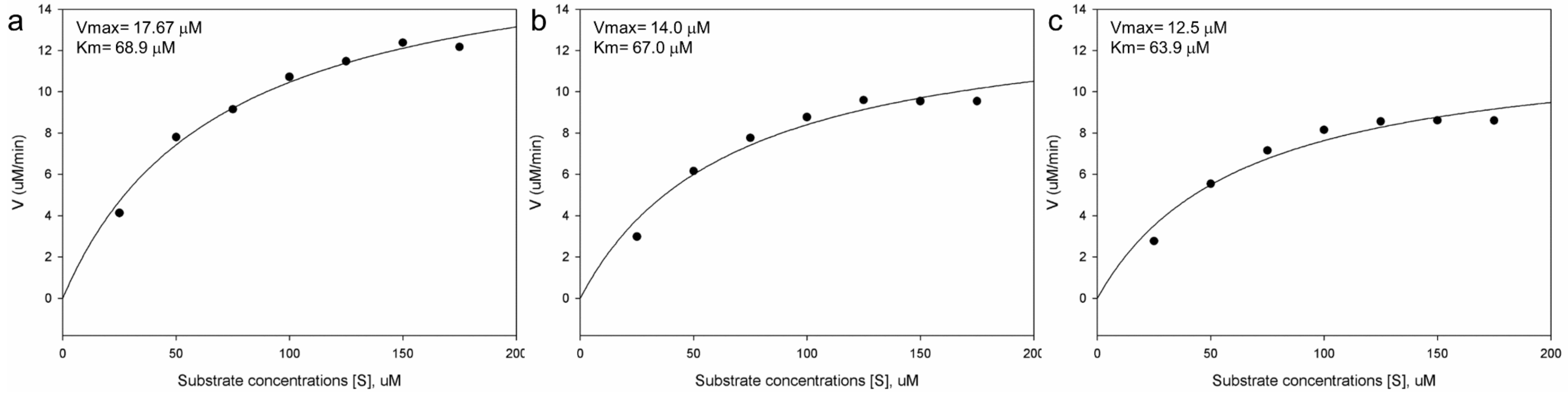

Fig. S5 The Michaelis-Menten plots. (a) Arachidonic acid, AA. (b) Eicosapentaenoic acid, EPA. (c) Docosahexaenoic acid, DHA.

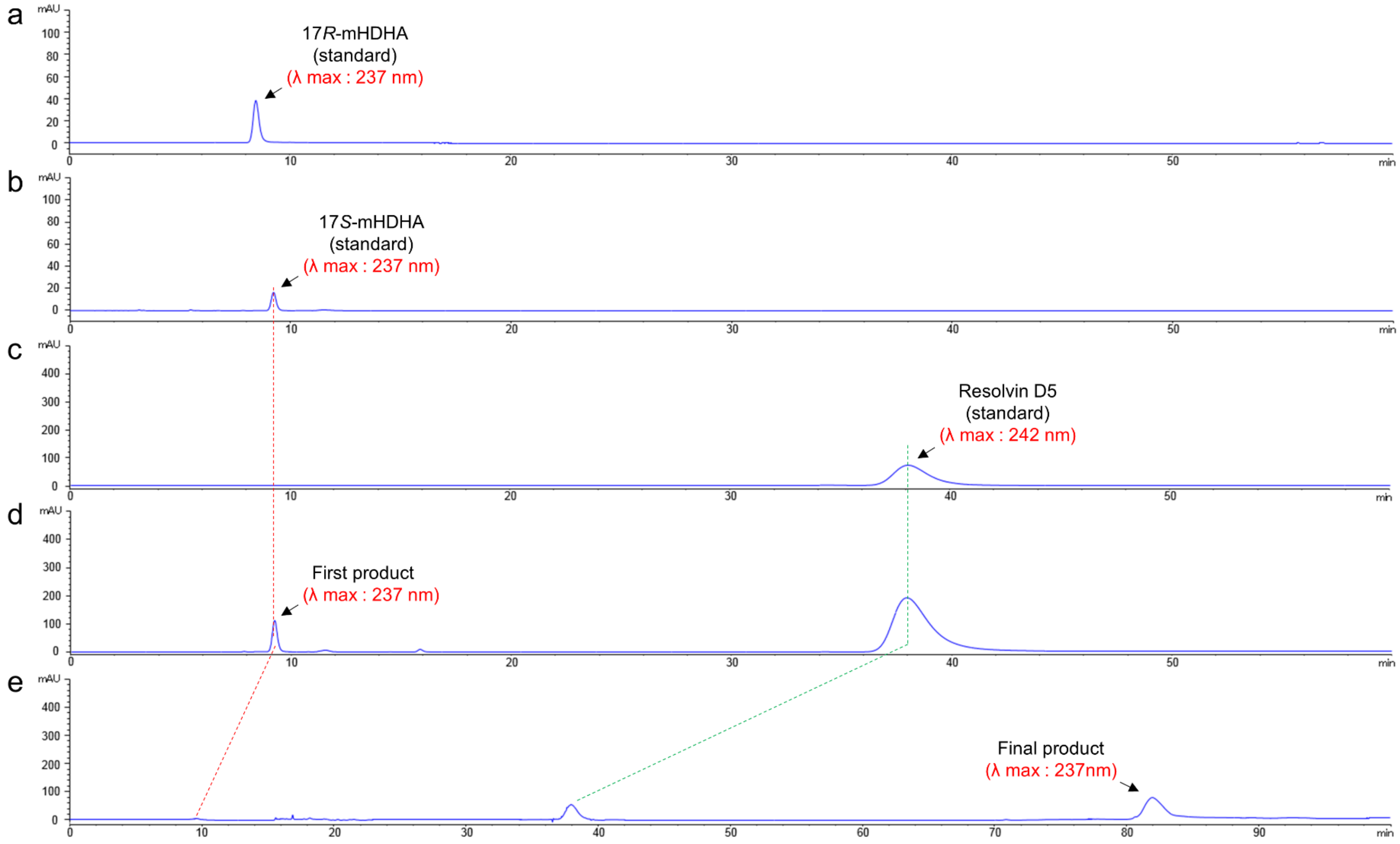

Fig. S6 CP-HPLC analysis for three products of Osc-LOX with DHA. (a) 17*R*-HDHA standard at 8.45 min. (b) 17*S*-HDHA standard at 9.22 min. (c) Resolvin D5 standard at 38 min. (d) The first product (17*S*-HDHA) and second product (RvD5) were generated from DHA by an Osc-LOX. (e) The final product (82min) was generated from RvD5 by an Osc-LOX.

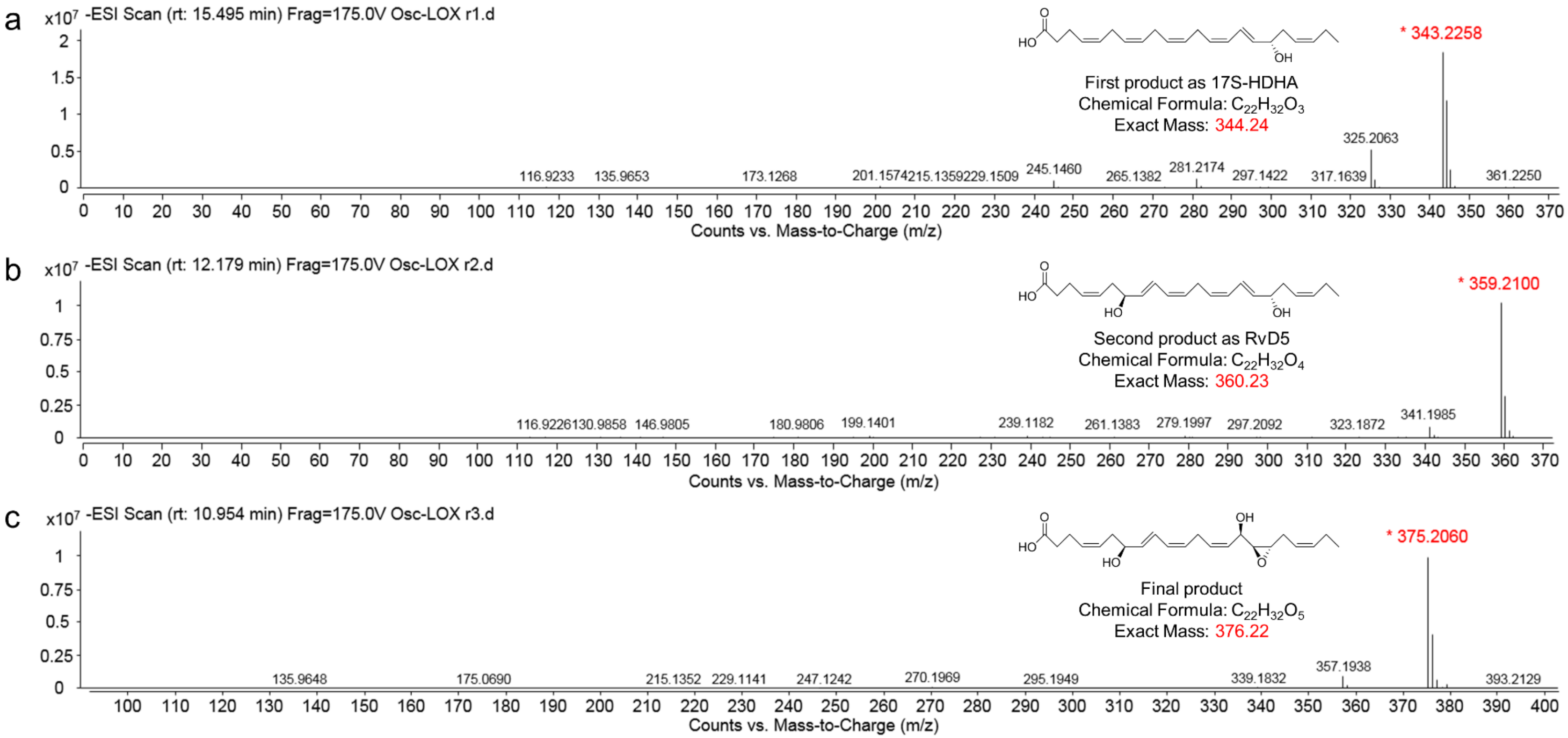

Fig. S7 LC-MS analysis of three products generated from DHA by Osc-LOX enzymatic catalysis. (a) 17*S*-HDHA, 17*S*-hydroxy-docosahexaenoic acid. (b) RvD5, 7*S*,17*S*-dihydroxy-docosahexaenoic acid. (c) Final product, 7*S*,15*R*-dihydroxy-16*S*,17*S*-epoxy-docosapentaenoic acid.

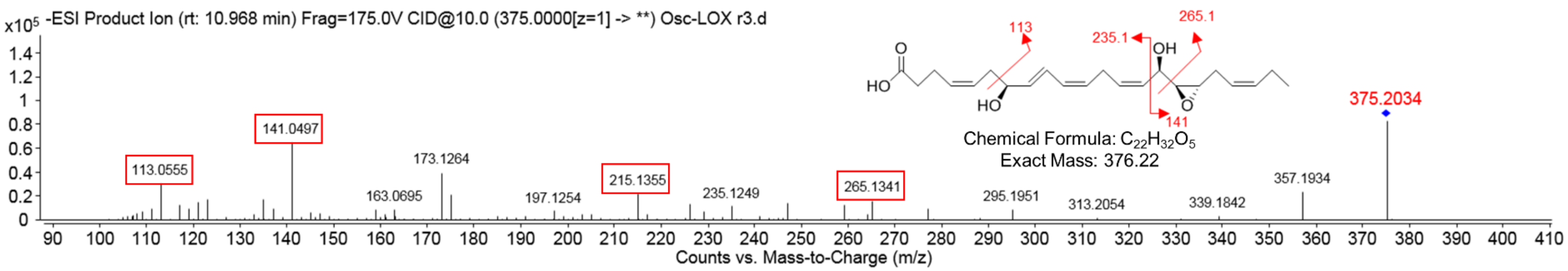

Fig. S8 LC-MS/MS analysis of 7*S*,15*R*-dihydroxy-16*S*,17*S*-epoxy-docosapentaenoic acid. This new product was generated from DHA via RvD5 by Osc-LOX. The blue rhombus square indicates total molecular mass of 7*S*,15*R*-dihydroxy-16*S*,17*S*-epoxy-docosapentaenoic acid and the empty red squares indicate molecular masses of fragments.

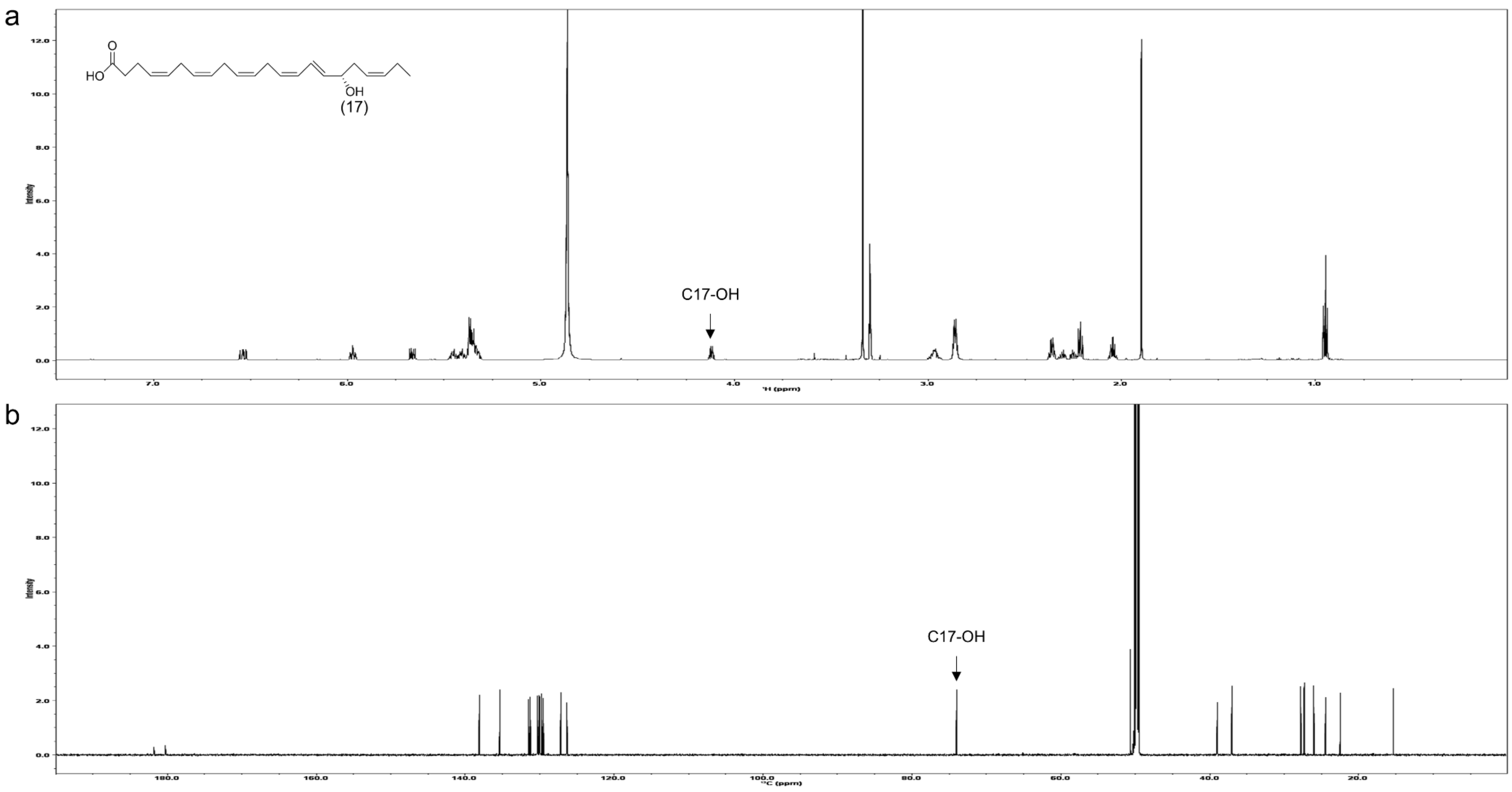

Fig. S9 1D NMR spectrum of 17*S*-HDHA. (a) 1H NMR peaks. (b) 13C NMR peaks.

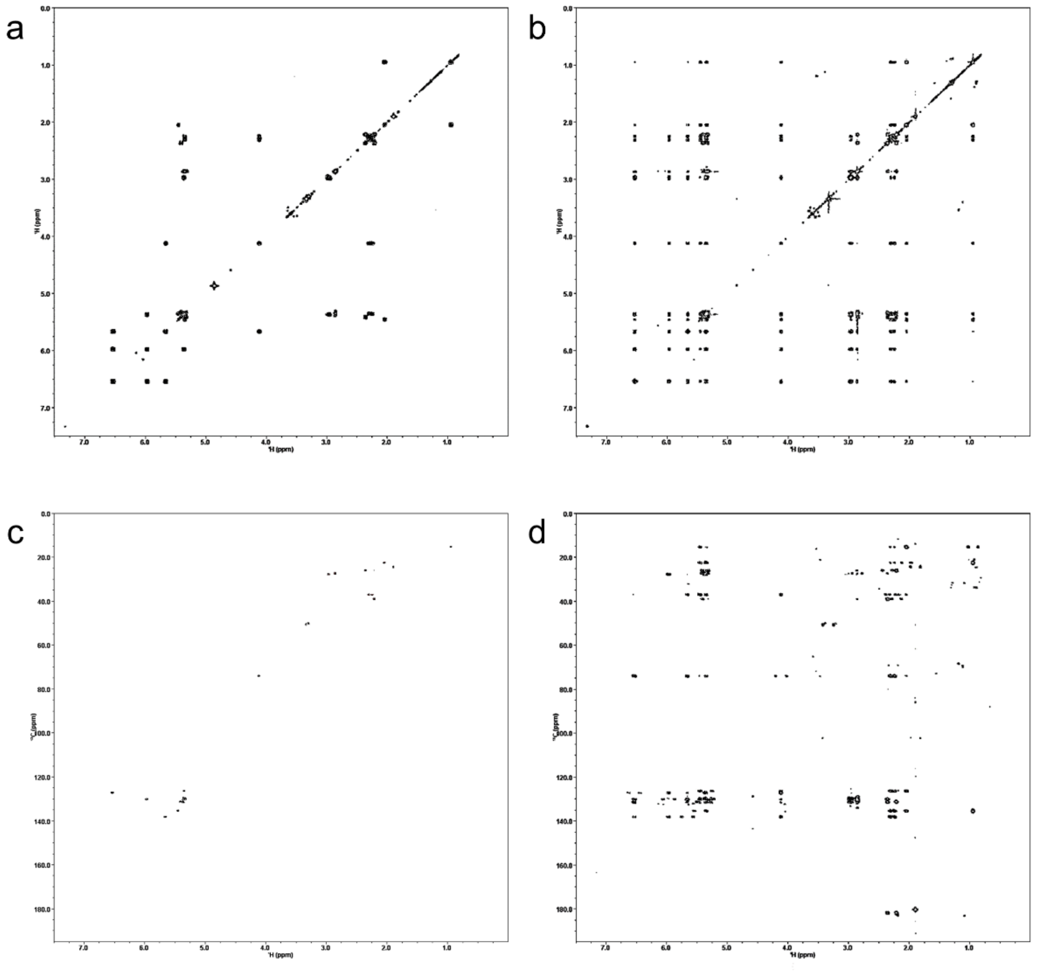

Fig. S10 2D NMR spectrum of 17*S*-HDHA. (a) COSY. (b) TOCSY. (c) HSQC. (d) HMBC.

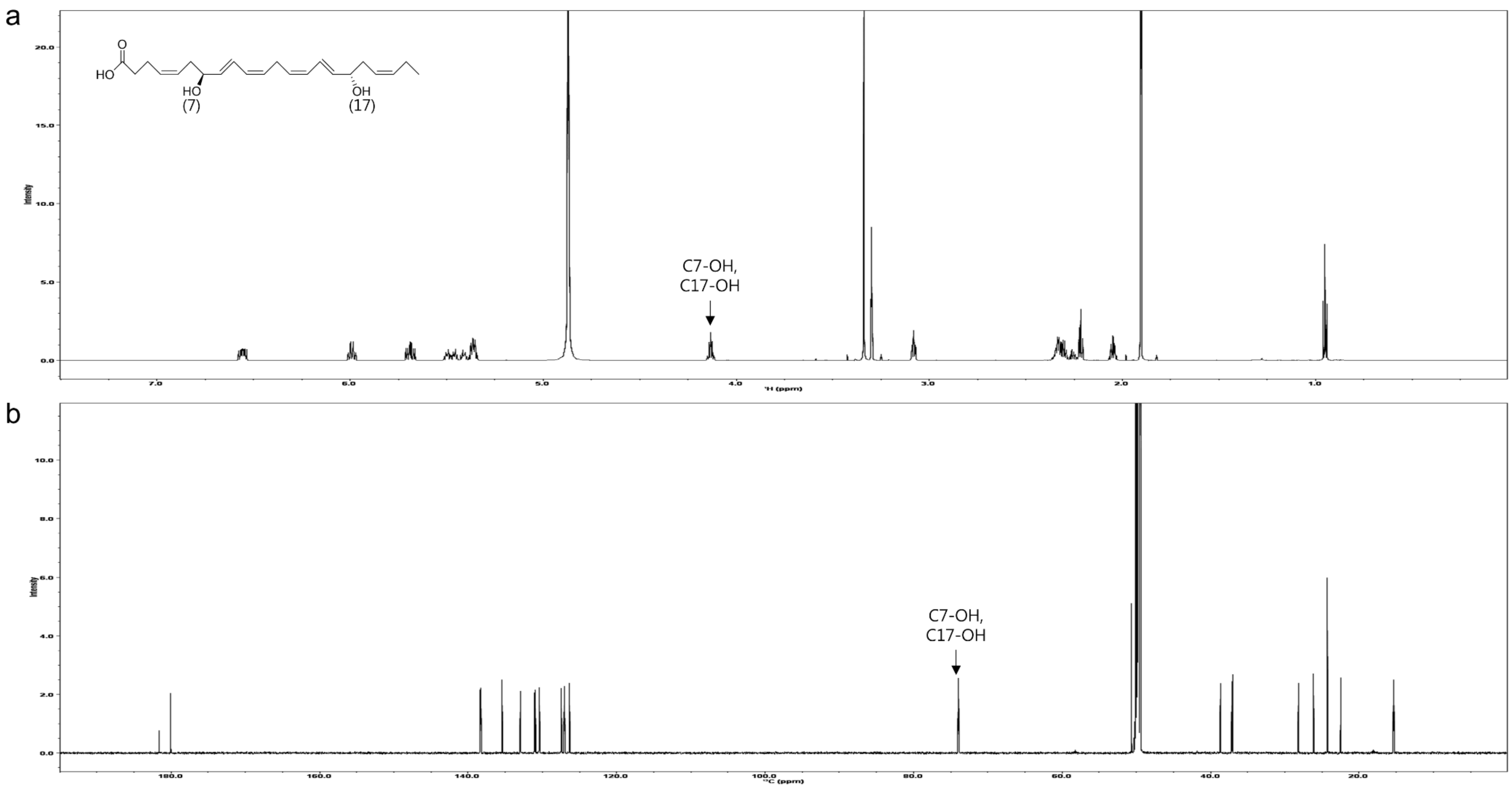

Fig. S11 1D NMR spectrum of resolvin D5 (RvD5). (a) 1H NMR peaks. (b) 13C NMR peaks.

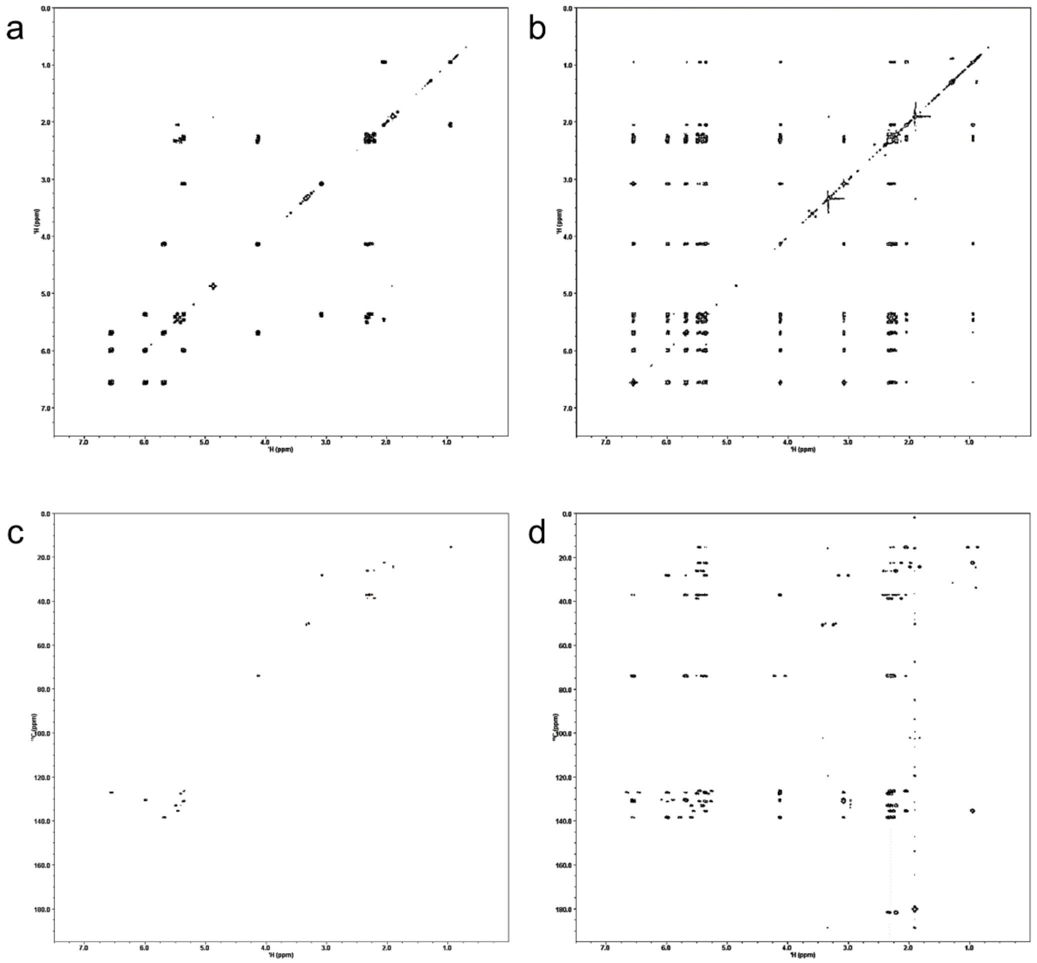

Fig. S12 2D NMR spectrum of resolvin D5 (RvD5). (a) COSY. (b) TOCSY. (c) HSQC. (d) HMBC.

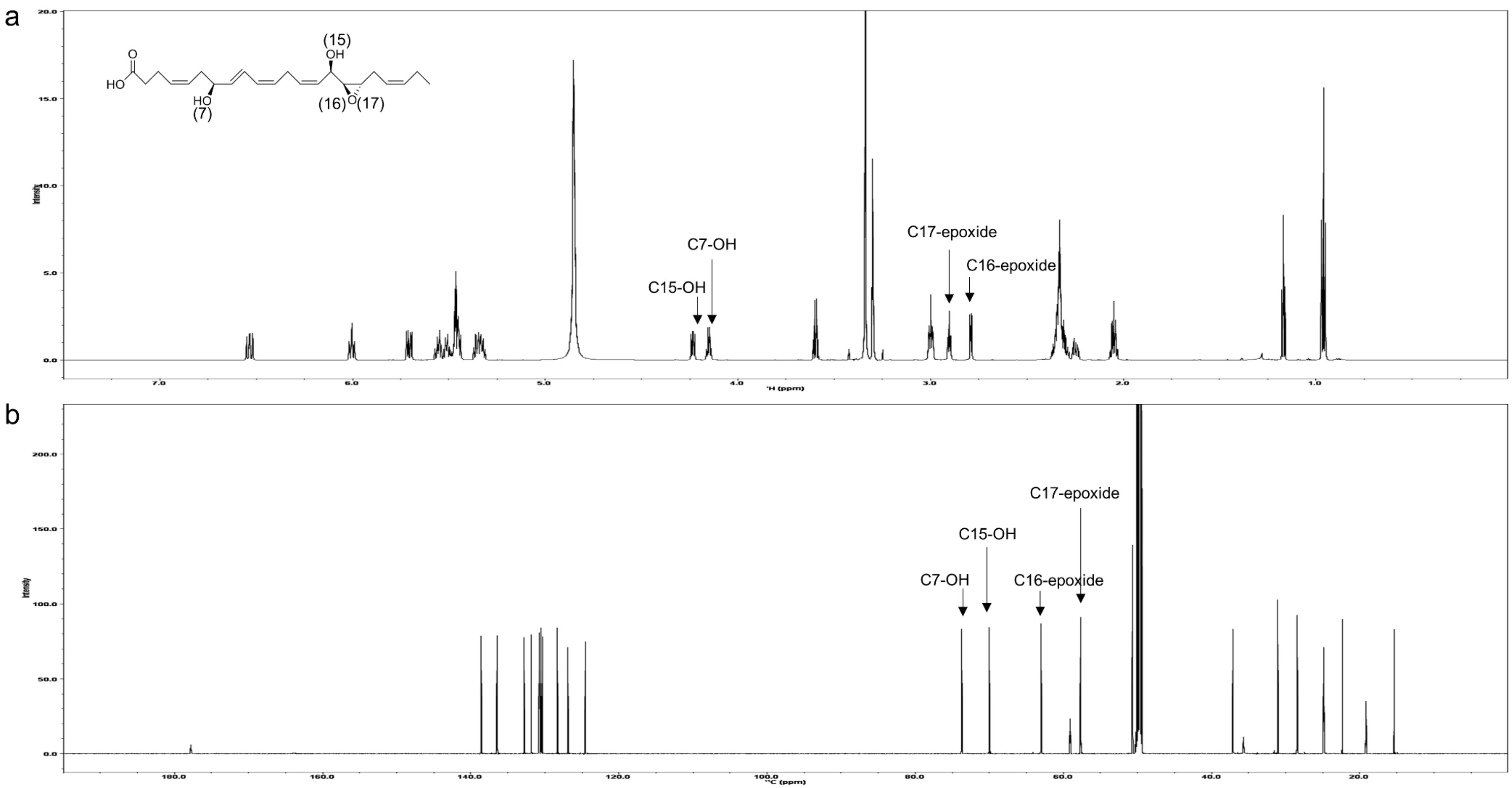

Fig. S13 1D NMR spectrum of 7*S*,15*R*-dihydroxy-16*S*,17*S*-epoxydocosa-4*Z*,8*E*,10*Z*,13*Z*,19*Z*-pentaenoic acid. (a) 1H NMR peaks. (b) 13C NMR peaks.

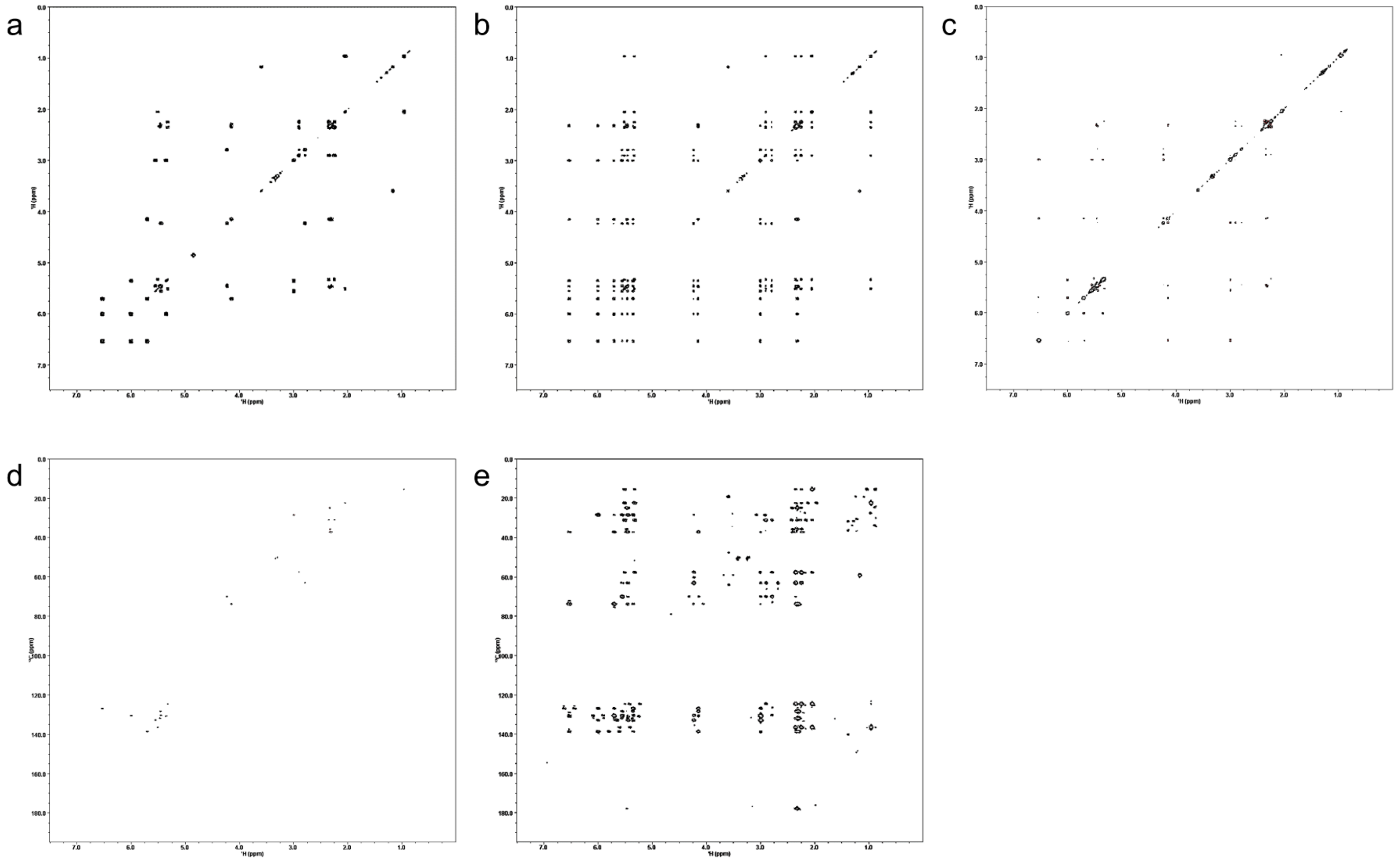

Fig. S14 2D NMR spectrum of 7*S*,15*R*-dihydroxy-16*S*,17*S*-epoxydocosa-4*Z*,8*E*,10*Z*,13*Z*,19*Z*-pentaenoic acid. (a) COSY. (b) TOCSY (c) NOESY. (d) HSQC. (e) HMBC.

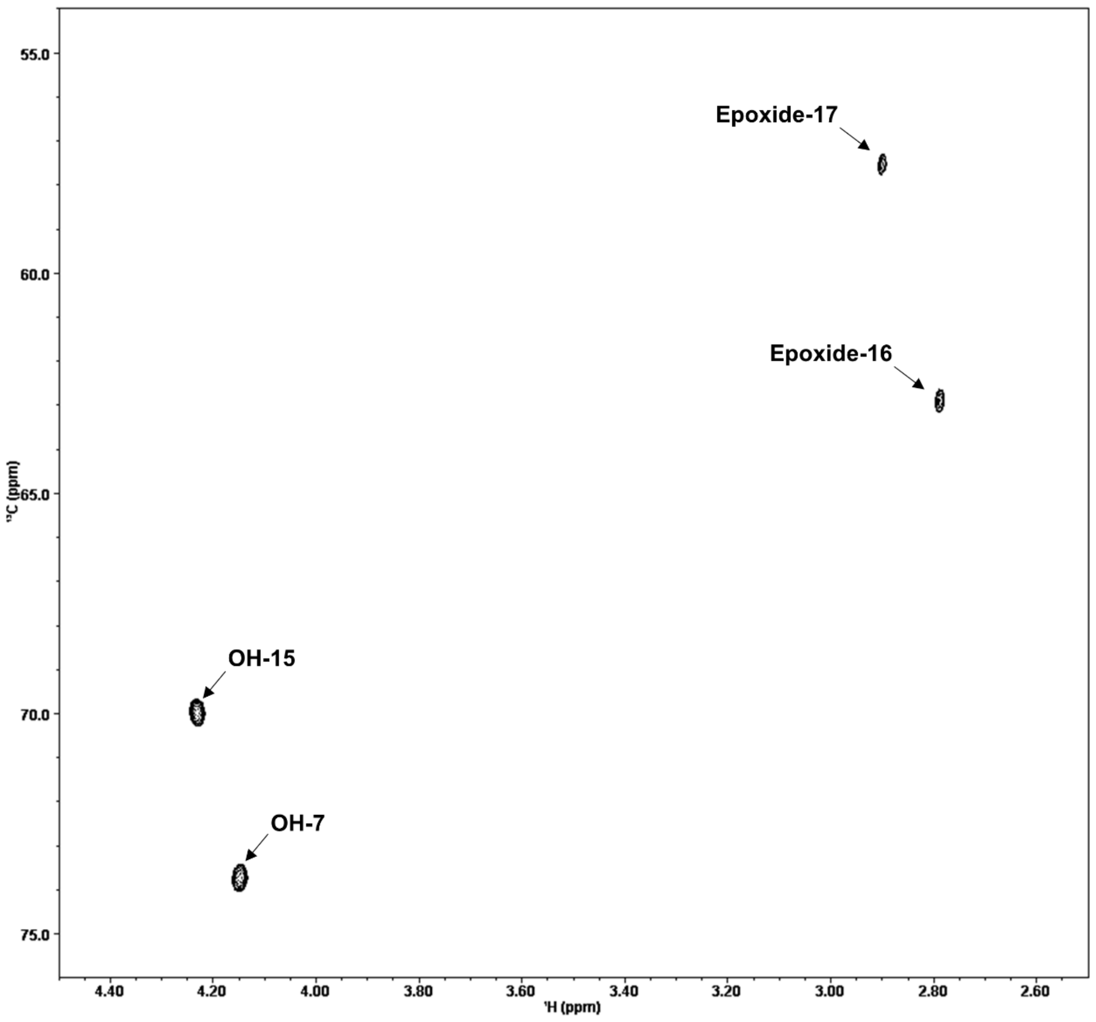

Fig. S15 HSQC spectrum of two hydroxyl groups and an epoxide group of 7*S*,15*R*-dihydroxy-16*S*,17*S*-epoxydocosa-4*Z*,8*E*,10*Z*,13*Z*,19*Z* -pentaenoic acid.

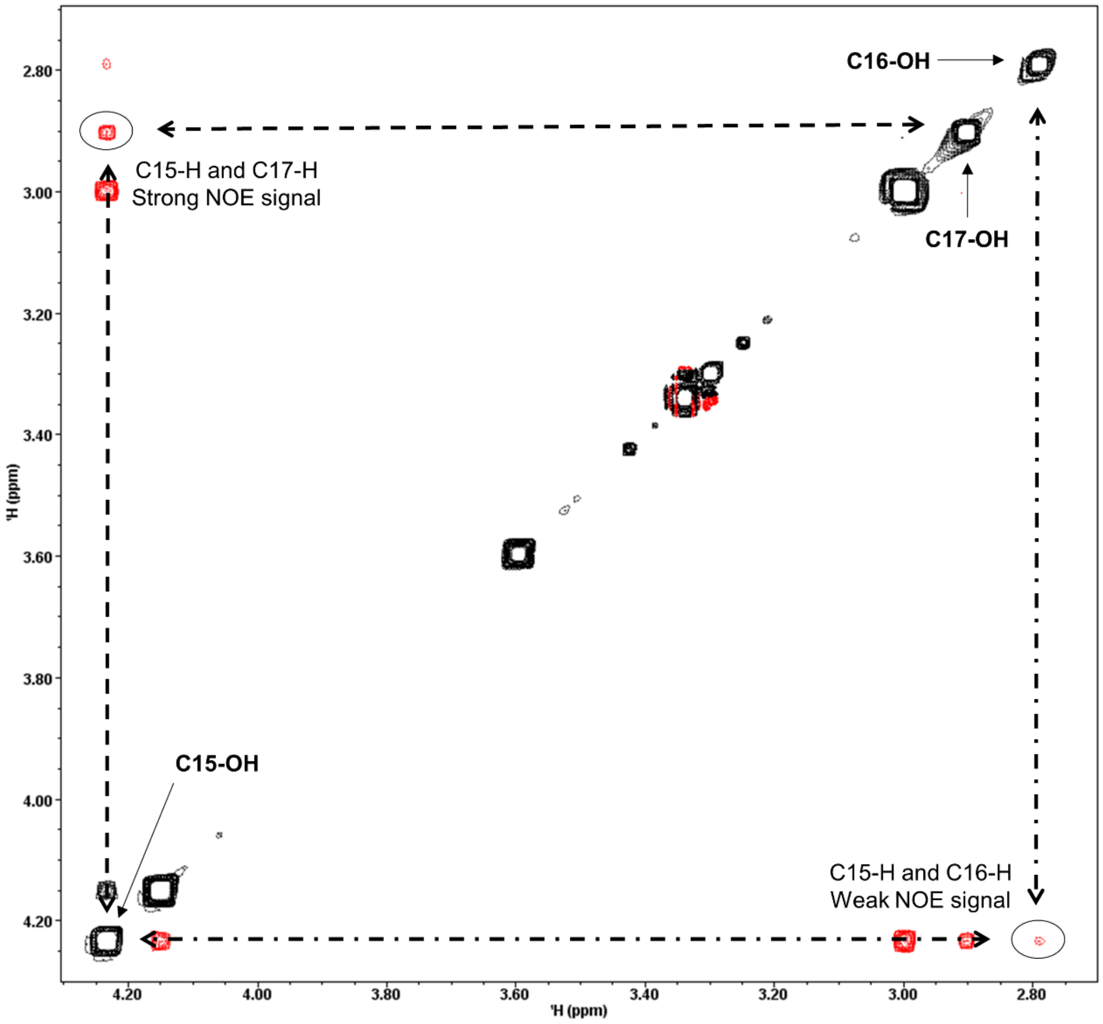

Fig. S16 NOESY spectrum of 7*S*,15*R*-dihydroxy-16*S*,17*S*-epoxydocosa-4*Z*,8*E*,10*Z*,13*Z*,19*Z*-pentaenoic acid.

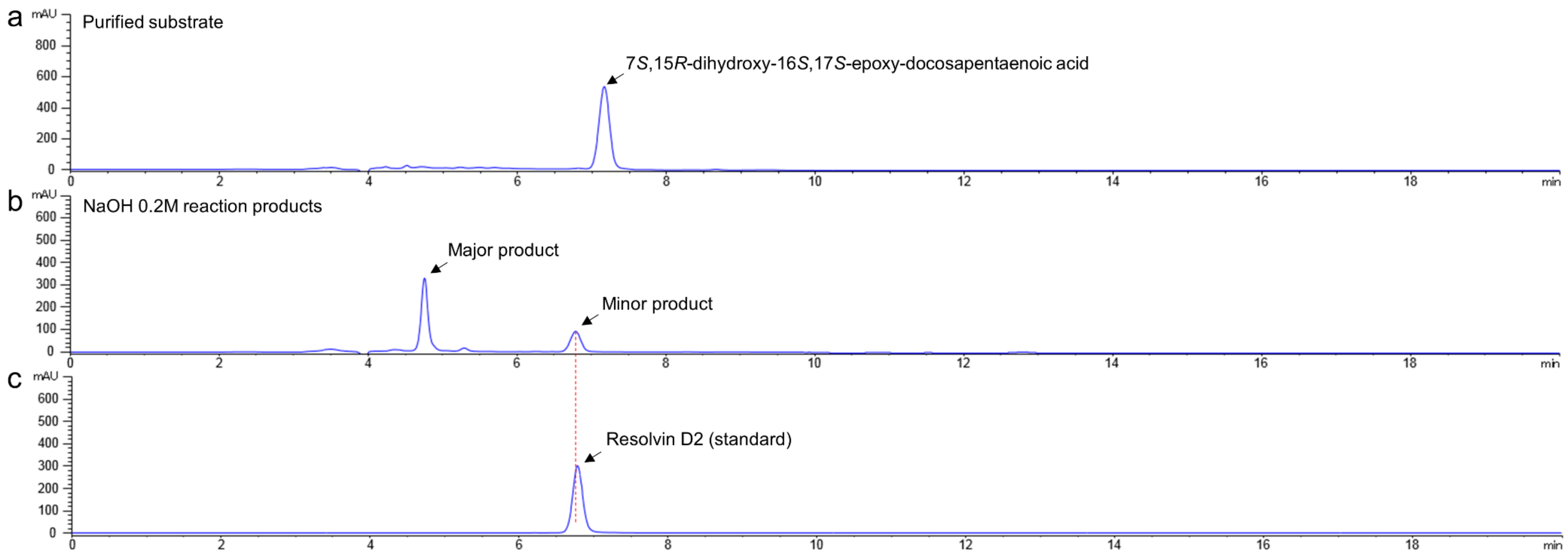

Fig. S17 RP-chiral HPLC analysis of epoxide ring-opened products by sodium hydroxide (NaOH). (a) Purified 7*S*,15*R*-dihydroxy-16*S*,17*S*-epoxy-docosapentaenoic acid as a precursor. (b) Two products were generated by NaOH: a major product was 7*S*,15*R*,16*S*,17*S*-tetrahydroxy docosapentaenoic acid and a minor product was RvD2. (c) RvD2 standard. The epoxide ring-opening reaction was performed with NaOH at 30 °C overnight.

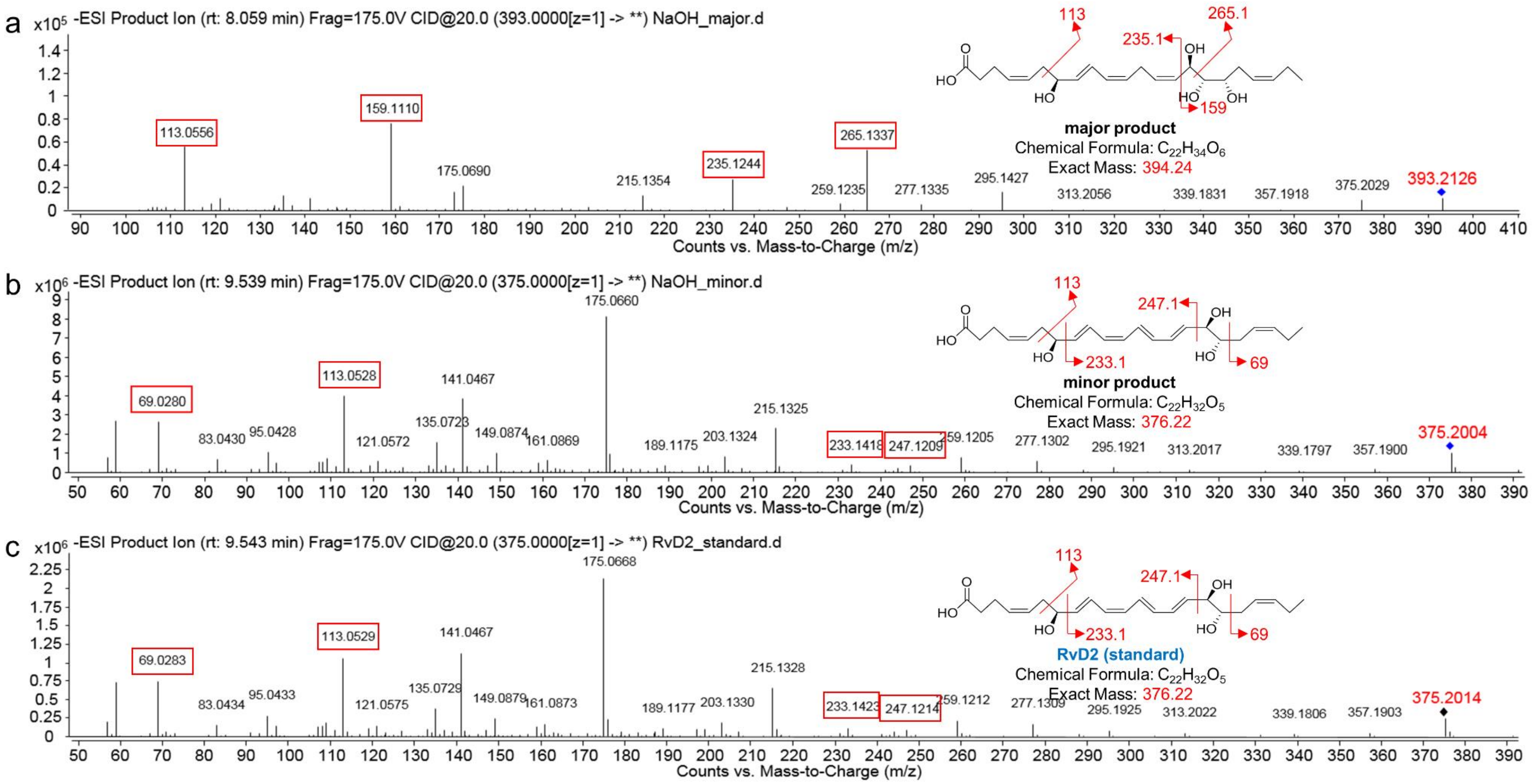

Fig. S18 LC-MS/MS analysis of 7*S*,15*R*,16*S*,17*S*-tetrahydroxy-4*Z*,8*E*,10*Z*,13*Z*,19*Z*-docosapentaenoic acid and RvD2. A 7*S*,15*R*-dihydroxy-16*S*,17*S*-epoxy-docosapentaenoic acid was converted by hydrolysis reaction using sodium hydroxide (NaOH) into 7*S*,15*R*,16*S*,17*S*-tetrahydroxy-4*Z*,8*E*,10*Z*,13*Z*,19*Z* -docosapentaenoic acid and resolvin D2. (a) Tetrahydroxy fatty acid, main product. (b) RvD2, minor product. (c) Commercially available standard RvD2. The blue rhombus square indicates total molecular mass and the empty red squares indicate molecular masses of fragments (a-c).

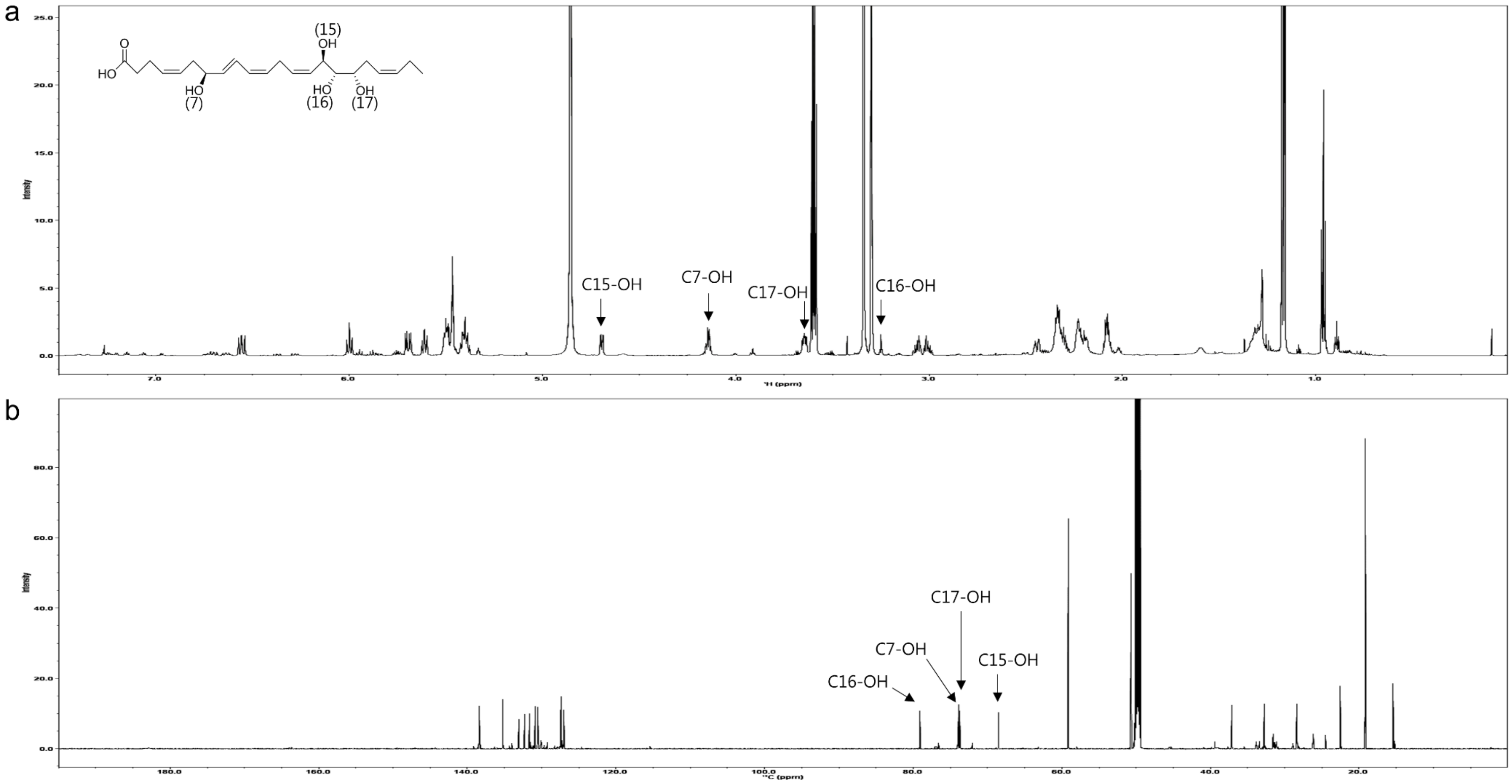

Fig. S19 1D NMR spectrum of 7*S*,15*R*,16*S*,17*S*-tetrahydroxy-4*Z*,8*E*,10*Z*,13*Z*,19*Z*-docosapentaenoic acid. (a) 1H NMR peaks. (b) 13C NMR peaks.

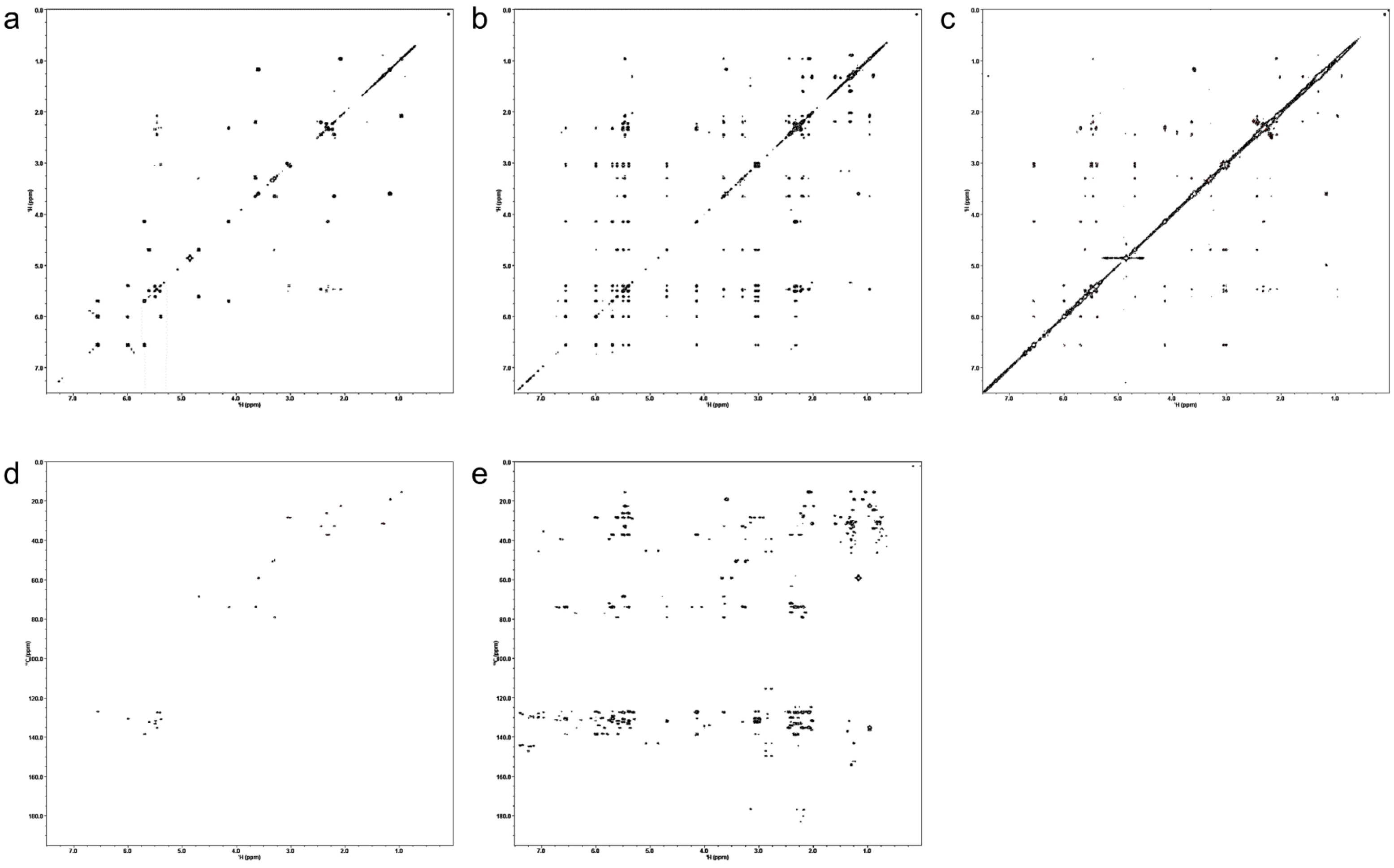

Fig. S20 2D NMR spectrum of 7*S*,15*R*,16*S*,17*S*-tetrahydroxy-4*Z*,8*E*,10*Z*,13*Z*,19*Z*-docosapentaenoic acid. (a) COSY. (b) TOCSY (c) NOESY. (d) HSQC. (e) HMBC.

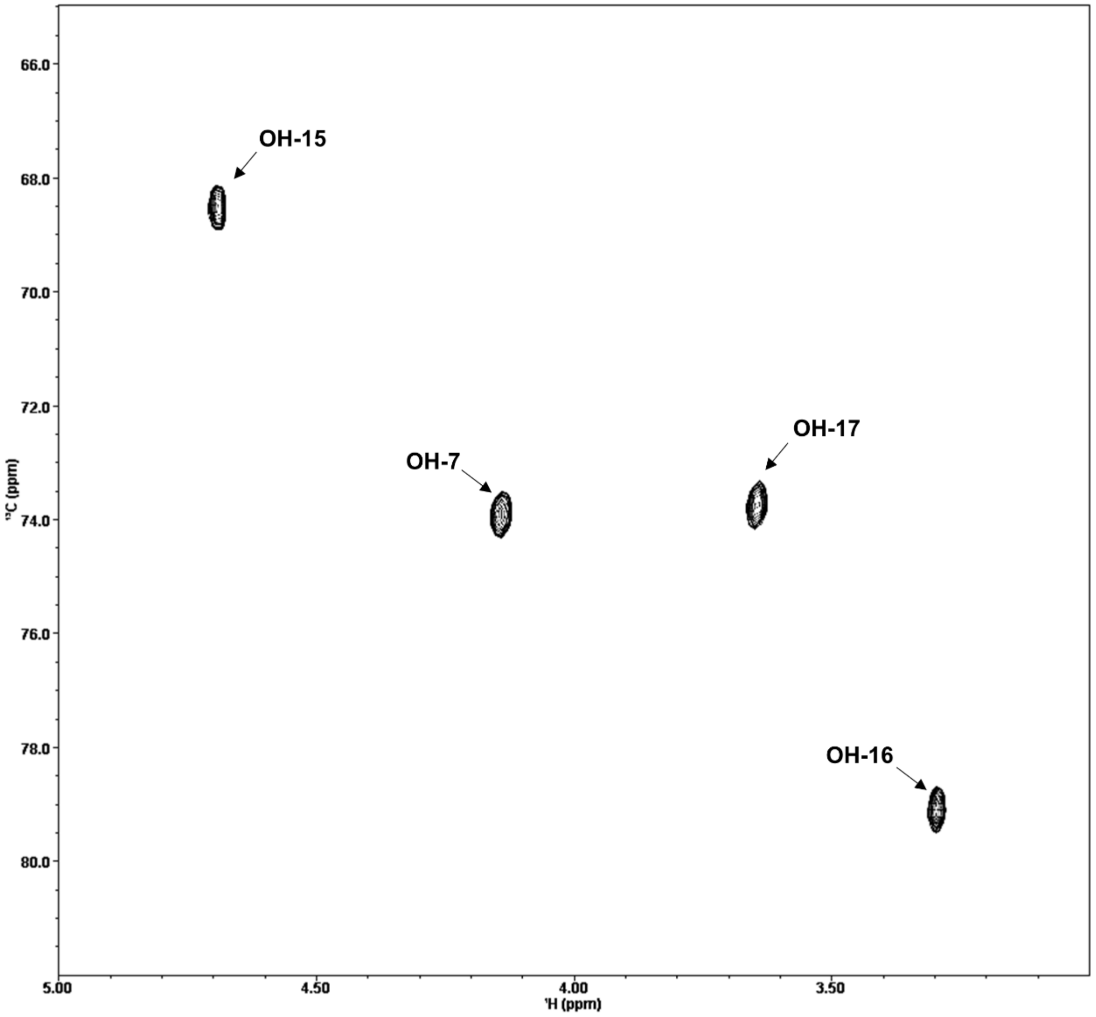

Fig. S21 HSQC spectrum of four hydroxyl groups of 7*S*,15*R*,16*S*,17*S*-tetrahydroxy-4*Z*,8*E*,10*Z*,13*Z*,19*Z*-docosapentaenoic acid.

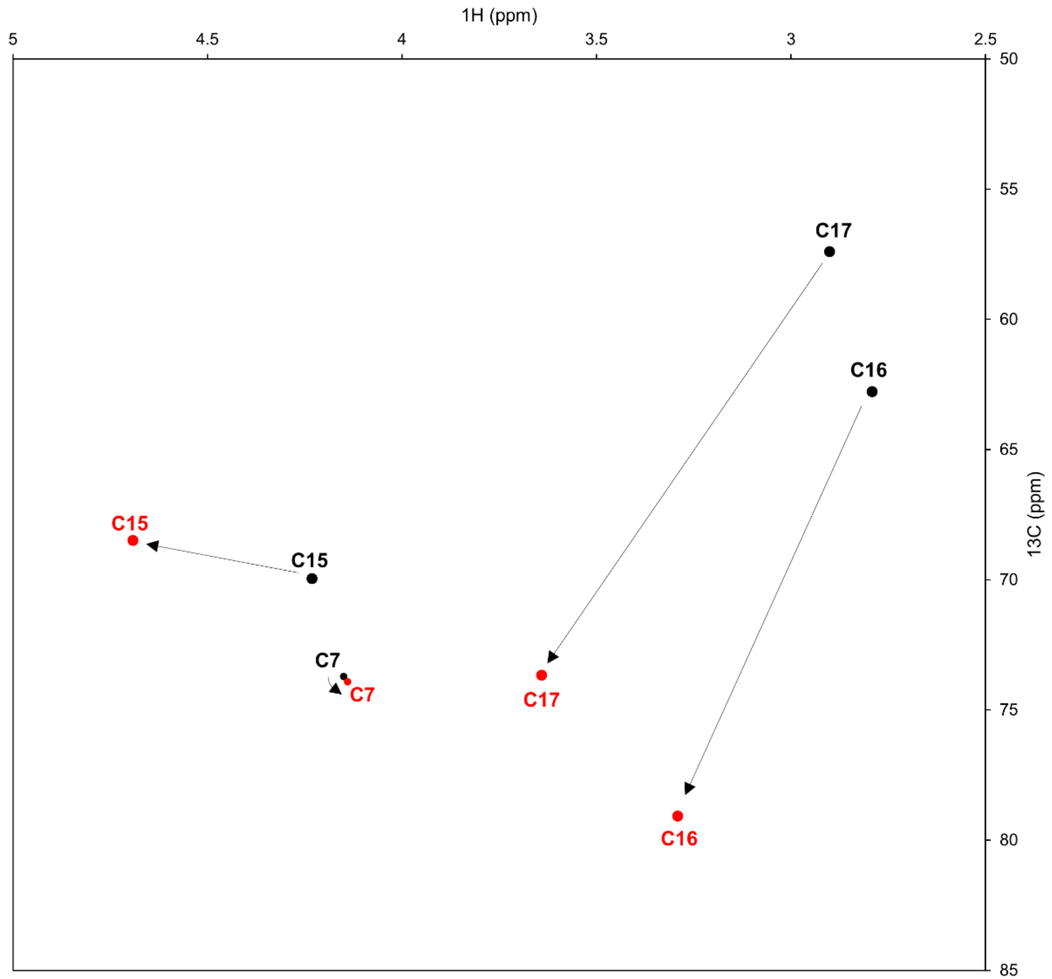

Fig. S22 Changed chemical shifts were monitored in HSQC spectrum. The black filled circles represent the peak of 7*S*,15*R*-dihydroxy-16*S*,17*S*-epoxy-docosapentaenoic acid that was used as precursor. The red filled circles indicate the peak of newly formed 7*S*,15*R*,16*S*,17*S*-tetrahydroxy-4*Z*,8*E*,10*Z*,13*Z*,19*Z*-docosapentaenoic acid by epoxide ring-opening.

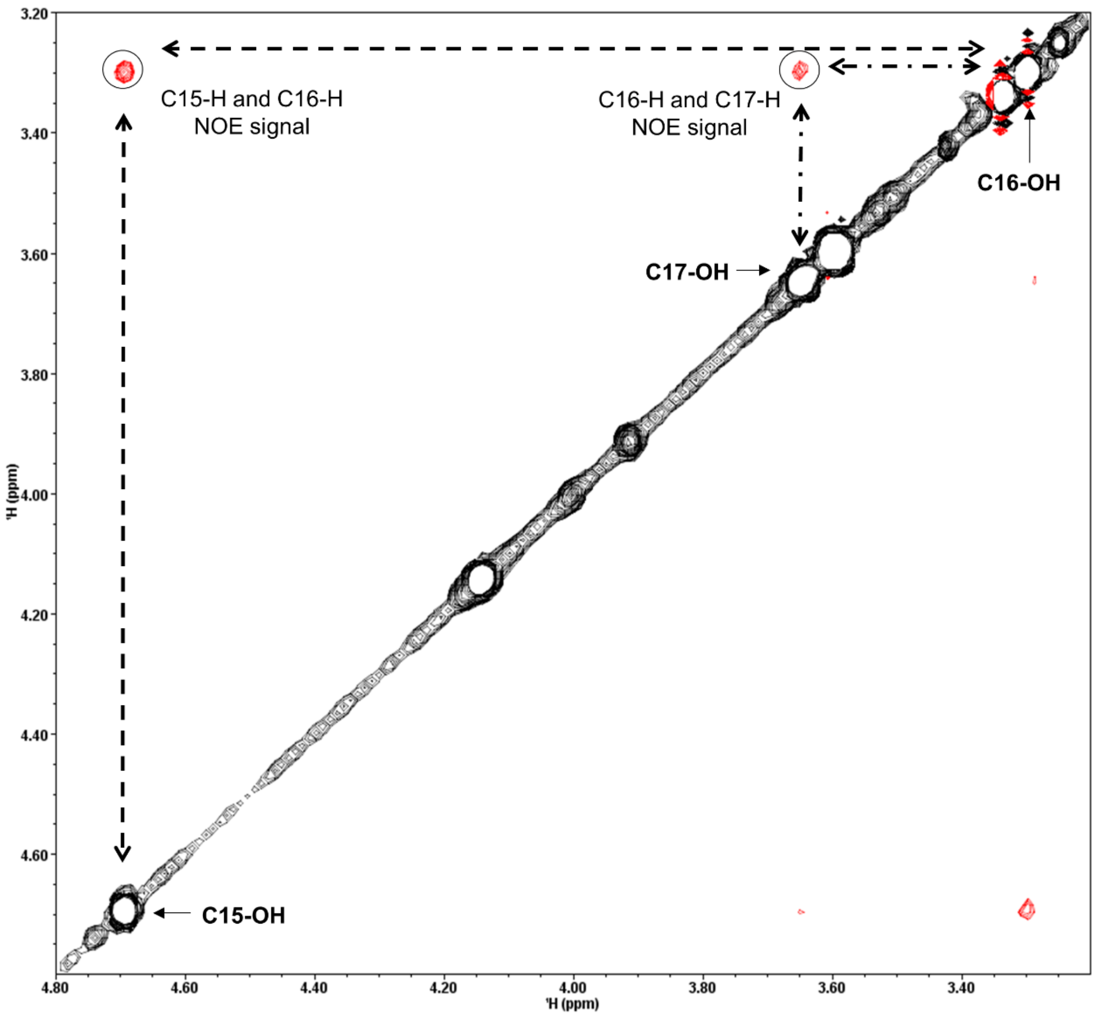

Fig. S23 NOESY spectrum of 7*S*,15*R*,16*S*,17*S*-tetrahydroxy-4*Z*,8*E*,10*Z*,13*Z*,19*Z* -docosapentaenoic acid.

Table S1. Oligonucleotide Primers of *osc-lox* for PCR.

Name	Enzyme	Primer sequence (5' → 3')
Osc-LOX (F)	*Nde* Ⅰ	CATATGGTAGACAATATGAAACCGTCTCTTC
Osc-LOX (R)	*Xho* Ⅱ	CTCGAGTTACATACTGATGCTATTGATTACC

Table S2. Enzymatic activity of Osc-LOX in each purification steps.

	**Crude fraction (After cell disruption)**	**1st purification**	**2nd purification**
Total activity (Killounit L^{-1})	146 (+/- 5)	104 (+/- 12)	40 (+/- 10)

Table S3. 1D NMR (1H and 13C) data of conversion products from DHA

Carbon number	(a) 17S-HDHA			(b) Resolvin D5; RvD5			(c) New lipid mediator 1[a]			(d) New lipid mediator 2[b]		
	13C (δ)	1H (δ)	Feature	13C (δ)	1H (δ)	Feature	13C (δ)	1H (δ)	Feature	13C (δ)	1H (δ)	Feature
1	181.81	-	COOH	181.63		COOH	177.77	-	COOH	176.8		COOH
2	39.01	2.21	CH2	38.68	2.21	CH2	35.69	2.33	CH2	26.2	2.23	CH2
3	26.04	2.36	CH2	26.11	2.33	CH2	24.8	2.33	CH2	37.11	2.32	CH2
4	131.25	5.41	CH	132.93	5.49	CH	131.79	5.47	CH	127.47	5.4	CH
5	130	5.33	CH	127.46	5.41	CH	128.34	5.46	CH	133.07	5.49	CH
6	27.32	2.86	CH2	37.1	2.33	CH2	37.07	2.33	CH2	26.19	2.33	CH2
7	129.55	5.36	CH	73.93, 73.96	4.13	OH	73.71	4.15	OH	73.91	4.14	OH
8	130	5.35	CH	138.4	5.7	CH	138.66	5.7	CH	138.41	5.69	CH
9	27.34	2.86	CH	127.01, 127.08	6.55	CH	126.89	6.54	CH	127.01	6.57, 6.55	CH
10	130.29, 130.34	5.36	CH	130.38-, 30.43	5.99	CH	130.53	6.01	CH	130.57	5.99	CH
11	130.29, 130.34	5.36	CH	130.96, 131.07	5.36	CH	130.75	5.35	CH	130.87	5.39	CH
12	27.79	2.97	CH2	28.17	3.08	CH2	28.43	3	CH2	28.36, 28.43	3.05, 3.01	CH2
13	131.43	5.36	CH	130.96, 131.07	5.36	CH	132.77	5.555	CH	131.66	5.49	CH
14	130.12	5.97	CH	130.38, 130.43	5.99	CH	130.33	5.45	CH	132.3	5.61	CH
15	127.18	6.54	CH	127.01, 127.08	6.55	CH	69.97	4.23	OH	68.51	4.69	OH
16	138.12	5.66	CH	138.31	5.69	CH	62.79	2.79	Epoxide	79.09	3.29	OH
17	73.99	4.12	OH	73.93, 73.96	4.13	OH	57.42	2.9	Epoxide	73.68	3.64	OH
18	37.05	2.3, 2.25	CH2	37.09	2.26	CH2	30.99, 31.04	2.34, 2.25	CH2	32.8	2.19, 2.4	CH2
19	126.35	5.35	CH	126.3	5.36	CH	124.52	5.33	CH	127.4	5.46	CH
20	135.4	5.45	CH	135.42	5.46	CH	136.44	5.51	CH	135.22	5.46	CH
21	22.5	2.04	CH2	22.46	2.05	CH2	22.35	2.05	CH2	22.5	2.08	CH2
22	15.32	0.95	CH3	15.33	0.95	CH3	15.35	0.96	CH3	15.4	0.96	CH3

[a] 7*S*,15*R*-dihydroxy-16*S*,17*S*-epoxydocosa-4*Z*,8*E*,10*Z*,13*Z*,19*Z*-pentaenoic acid.

[b] 7*S*,15*R*,16*S*,17*S*-tetrahydroxy-4*Z*,8*E*,10*Z*,13*Z*,19*Z*-pentaenoic acid.

Table S4. List of conversion products of DHA.

Product	Formal name
17*S*-HDHA	17*S*-hydroxy-4*Z*,7*Z*,10*Z*,13*Z*,15*E*,19*Z*-docosahexaenoic acid
RvD2	7*S*,16*R*,17*S*-trihydroxy-4*Z*,8*E*,10*Z*,12*E*,14*E*,19*Z*-docosahexaenoic acid
RvD5	7*S*,17*S*-dihydroxy-4*Z*,8*E*,10*Z*,13*Z*,15*E*,19*Z*-docosahexaenoic acid
New lipid mediator 1	7*S*,15*R*-dihydroxy-16*S*,17*S*-epoxydocosa-4*Z*,8*E*,10*Z*,13*Z*,19*Z*-pentaenoic acid
New lipid mediator 2	7*S*,15*R*,16*S*,17*S*-tetrahydroxydocosa-4*Z*,8*E*,10*Z*,13*Z*,19*Z* -pentaenoic acid
